# Hierarchical biota-level and taxonomic controls on the chemistry of fossil melanosomes revealed using synchrotron X-ray fluorescence

**DOI:** 10.1038/s41598-020-65868-3

**Published:** 2020-06-02

**Authors:** Valentina Rossi, Samuel M. Webb, Maria E. McNamara

**Affiliations:** 1 School of Biological, Earth and Environmental Sciences, University College Cork, North Mall, Cork, T23 TK30 Ireland; 2 Stanford Synchrotron Radiation Lightsource (SSRL), SLAC National Accelerator Laboratory, Menlo Park, CA 94025 USA

**Keywords:** Evolution, Biogeochemistry

## Abstract

Fossil melanosomes, micron-sized granules rich in melanin *in vivo*, provide key information for investigations of the original coloration, taxonomy and internal anatomy of fossil vertebrates. Such studies rely, in part, on analysis of the inorganic chemistry of preserved melanosomes and an understanding of melanosome chemical taphonomy. The extent to which the preserved chemistry of fossil melanosomes is biased by biotic and abiotic factors is, however, unknown. Here we report the discovery of hierarchical controls on the inorganic chemistry of melanosomes from fossil vertebrates from nine biotas. The chemical data are dominated by a strong biota-level signal, indicating that the primary taphonomic control is the diagenetic history of the host sediment. This extrinsic control is superimposed by a biological, tissue-level control; tissue-specific chemical variation is most likely to survive in fossils where the inorganic chemistry of preserved melanosomes is distinct from that of the host sediment. Comparative analysis of our data for fossil and modern amphibians reveals that most fossil specimens show tissue-specific melanosome chemistries that differ from those of extant analogues, strongly suggesting alteration of original melanosome chemistry. Collectively, these findings form a predictive tool for the identification of fossil deposits with well-preserved melanosomes amenable to studies of fossil colour and anatomy.

## Introduction

Melanins are a family of chemically diverse pigments that are widespread in organisms from all kingdoms of life^1^ and occur either disseminated through body tissues with no morphological expression (e.g. as in arthropod cuticle^2^) or as discrete membrane-bound organelles, i.e. melanosomes (as in vertebrates^3^). Melanins have fundamental physiological roles, including coloration^1^, prevention of UV^4^ and oxidative^5^ damage and as a sink for metals^1^.

It is well established that melanin and melanosomes can preserve within the integumentary tissues (i.e. skin, feathers and hair) of fossil vertebrates, often as organic remains^6–17^. Arguments that preserved melanosomes are instead fossil bacteria^18,19^ are untenable given the abundant chemical evidence for degraded or intact melanin associated with melanosome-like microbodies in fossils^17,20–23^, because bacteria are not known to preserve as organic remains outside conservation media such as amber or chert, and given the lack of a taphonomic mechanism to explain such preservation. Recent developments in the study of fossil melanin and melanosomes have provided unprecedented insights into various aspects of animal biology and evolution, including the colour^6–11^, behaviour^12,13^, phylogenetic affinities^14,15^ and internal anatomy^16,17^ of ancient animals.

Evidence of melanin and melanosomes has been reported from fossils that vary in taxonomy, age and geological setting^14,15,22,24^; this broad distribution in the fossil record provides an excellent opportunity to investigate the controls on melanin and melanosome taphonomy. Most studies have focussed on integumentary melanosomes, especially those from feathers, which can preserve as three-dimensionally preserved microbodies^6^ or as external moulds^7,25^. Recent experiments have shown that preservation of melanosomes as moulds can result from reaction with an oxidant, resulting in dissolution of melanosomes^26^. Other experimental studies using feather melanosomes have shown that elevated pressures and temperatures can result in melanosome shrinkage by *ca*. 10–20% of the original size^27,28^. Thermal maturation experiments have also been applied to studies of the molecular taphonomy of melanin; the observed increase in cross-linking among melanin molecular units is considered to enhance the preservation potential of melanin^22,29,30^. Another important taphonomic factor is the strong affinity of melanin for metals (reviewed in ref. ^31^). Incorporation of metals^32^ and other elements, e.g. S^32^, during fossilization can promote the preservation of melanosomes via enhanced molecular cross-linking^23^.

The association between melanosomes and metals in extant animals is well known^31,33,34^. Our pilot study of a limited number of fossil vertebrates^17^ showed that tissue-specific metal-melanosome associations are evident in some fossils. Such associations also characterise extant analogues and have been interpreted as evidence for functions of melanin in metal homeostasis^17^. Preservation of tissue-specific chemistries in fossils (which is difficult to explain as a wholly diagenetic phenomenon) therefore suggests that at least a component of original chemistry, and functions of melanin in metal regulation, may originate in deep time. Tissue-specific metal-melanosome associations have the potential to be a potent tool in palaeobiology for inferring fossil anatomy and the functional evolution of melanin, but only if biological and taphonomic components of the preserved chemical signature can be discriminated. Even where melanosomes from different tissues in fossil vertebrates are chemically distinct, the former typically show concentrations of metals (e.g., Cu, Zn, Ca, Fe, Ti, Mg and Ni) that are markedly higher than those in extant vertebrates^10,17,35^. This chemical enrichment has been attributed to an increase in the original concentrations of elements (i.e., present in the tissues *in vivo*) via desiccation during fossilization^10^. Alternatively, elevated concentrations of elements in fossil melanosomes may reflect the incorporation of extrinsic elements (e.g. from pore fluids) during diagenesis^17^. These contrasting hypotheses have not been tested and thus the controls on the inorganic chemistry of fossil melanosomes are not fully resolved.

**Figure 1:**
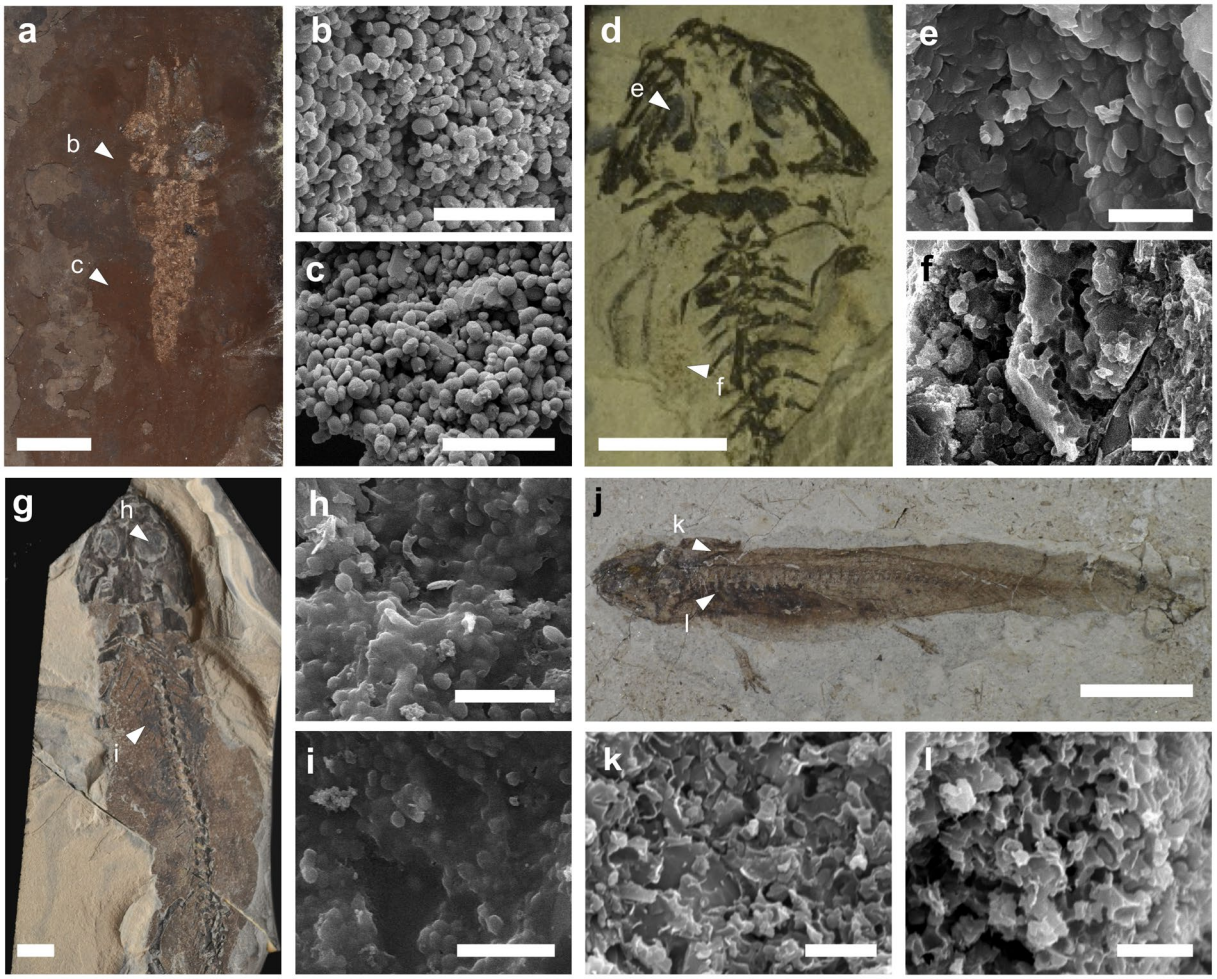
Fossil vertebrates and preserved melanosomes. (**a**,**d**,**g**,**j**) Fossil specimens presenting dark organic rich films. Scale bars 10 mm. (**a**) *P. diluvianus* (NHML-30271); (**d**) *B. amplystomus* (NHMD-155208); (**g**) *M. credneri* (NHMB-MB-Am.-1187); (**j**) Amphibia indet. (CNU-SAL-NN2013002P). White triangles denote sampling points. (**b**,**c**,**e**,**f**,**h**,**i**,**k**,**l**) Scanning electron micrographs of preserved melanosomes in soft tissue samples. Scale bars: **b**,**c**,**e**,**f**,**g**,**i**, 5 µm;  **k** and **l**, 2 µm.

Here, we test the fidelity of preservation of the inorganic chemistry of fossil melanosomes in the fossil record using 116 samples of melanosome-rich soft tissues (and associated sedimentary matrix) from 21 fossil vertebrates from nine biotas that range in age from the Early Permian to the Late Miocene (Table 1). All specimens show macroscopic evidence for soft tissue features defined as dark organic films, including some, or all, of the following: eyespots, the body outline (interpreted as skin), external gills and various features in the torso (Fig. 1). Synchrotron rapid scanning-X-ray fluorescence analysis (SRS-XRF) reveals a broad spectrum in melanosome chemistry: fossils from some, mostly Cenozoic, biotas retain strong tissue-specific melanosome chemistries that are distinct to the host sediment, but other, mostly Mesozoic and Palaeozoic, fossils show more homogenous melanosome chemistries that closely resemble that of the sedimentary matrix and are thus likely diagenetic in origin. Even where fossil melanosomes retain tissue-specific metal inventories, however, the latter differ from those in modern melanosomes. Fossil melanosome chemistry is thus subject to hierarchical controls, whereby for most biotas analysed, dominant diagenetic controls (linked in part to geological age) are superimposed by tissue-specific controls. These findings serve as a model for the preservation of tissue-specific chemical signatures in fossil melanosomes and as a new predictive tool to identify fossil biotas most amenable to studies of fossil melanosome chemistry.

**Table 1:** List of fossil specimens analysed. MSG is an abbreviation for Monte San Giorgio.

Specimen #	Class	Species name	Biota	Age	XRF
NHML-4999	Amphibia	*P. pueyoi*	Libros	Late Miocene	ref. ^17^
NHML-4982	Amphibia	*P. pueyoi*	Libros	Late Miocene	ref. ^17^
NHMB-MB.Am. 908	Amphibia	*Eopelobates sp*.	Orsberg	Miocene	SI
NHML-30271	Amphibia	*P. diluvianus*	Rott	Lower Miocene	SI
NHML-35814	Amphibia	*P. luedecki*	Bohemia	Oligo-Miocene	SI
HMLD-Me7069	Mammalia	Chiroptera indet.	Messel	Eocene	ref. ^17^
HLMD-Me5472	Aves	Aves indet.	Messel	Eocene	SI
IVPP-V13314	Aves	Aves indet.	Jehol	Cretaceous	in prep.
IVPP-V18357	Aves	Aves indet.	Jehol	Cretaceous	in prep.
IVPP-STM36-86	Reptilia	Reptilia indet.	Jehol	Cretaceous	in prep.
IVPP-STM36-2	Reptilia	Reptilia indet.	Jehol	Cretaceous	in prep.
IVPP-STM5-12	Reptilia	Dromeosauridae	Jehol	Cretaceous	in prep.
IVPP-STM9-5	Aves	*Yanornis*	Jehol	Cretaceous	in prep.
IVPP-V16525	Aves	*?Protopterix*	Jehol	Cretaceous	in prep.
CNU-VER-LB2009001	Reptilia	Squamata indet.	Yanliao	Jurassic	SI
CNU-SAL-NN2013002P	Amphibia	Urodela indet.	Yanliao	Jurassic	SI
PIMUZ-T3412	Reptilia	*N. peyeri*	MSG	Triassic	SI
PIMUZ-T3749	Reptilia	*N. edwardsii*	MSG	Triassic	SI
NHMB-MB.Am.1220	Amphibia	*A. pedestris*	Saar-Nahe	Permian	SI
NHMD-155208	Amphibia	*B. amplystomus*	Saar-Nahe	Permian	SI
NHMB-MB.Am.1187	Amphibia	*M. credneri*	Saar-Nahe	Permian	SI

## Results

### Fossil melanosomes vary in their mode of preservation

Melanosomes are preserved in all body regions analysed from all specimens (Fig. 1a–l, Supplementary Figs. S1–S6). The mode of preservation of melanosomes varies among specimens from different biotas and, less commonly, among regions of the body of individual specimens. In specimens from Cenozoic biotas, melanosomes are preserved as well-defined, three-dimensional microbodies with little or no associated organic matrix (Fig. 1a–c, Supplementary Figs. S1, S2). In older specimens, melanosomes are often poorly defined and surrounded by an organic matrix (Fig. 1d–i and Supplementary Figs. S3–S5). In some specimens, e.g. the Yanliao salamander (CNU-SAL-NN2013002P, Fig. 1j–l), melanosomes are preserved as moulds. Melanosomes in the fossil bird from Messel (HLMD-Me5472, Supplementary Fig. S6) exhibit distinct rod-like geometries similar to some feather melanosomes in extant and other fossil birds^17^ but different from the spherical to ovoid geometries of internal melanosomes in extant and fossil vertebrates^16,17^. The melanosomes of the fossil bird are thus likely to derive from feathers only. In all other specimens, melanosomes have ovoid to spherical geometries typical of melanosomes from the skin and internal organs in extant vertebrates^17^.

### Inter-biota variations in melanosome chemistry

**Figure 2:**
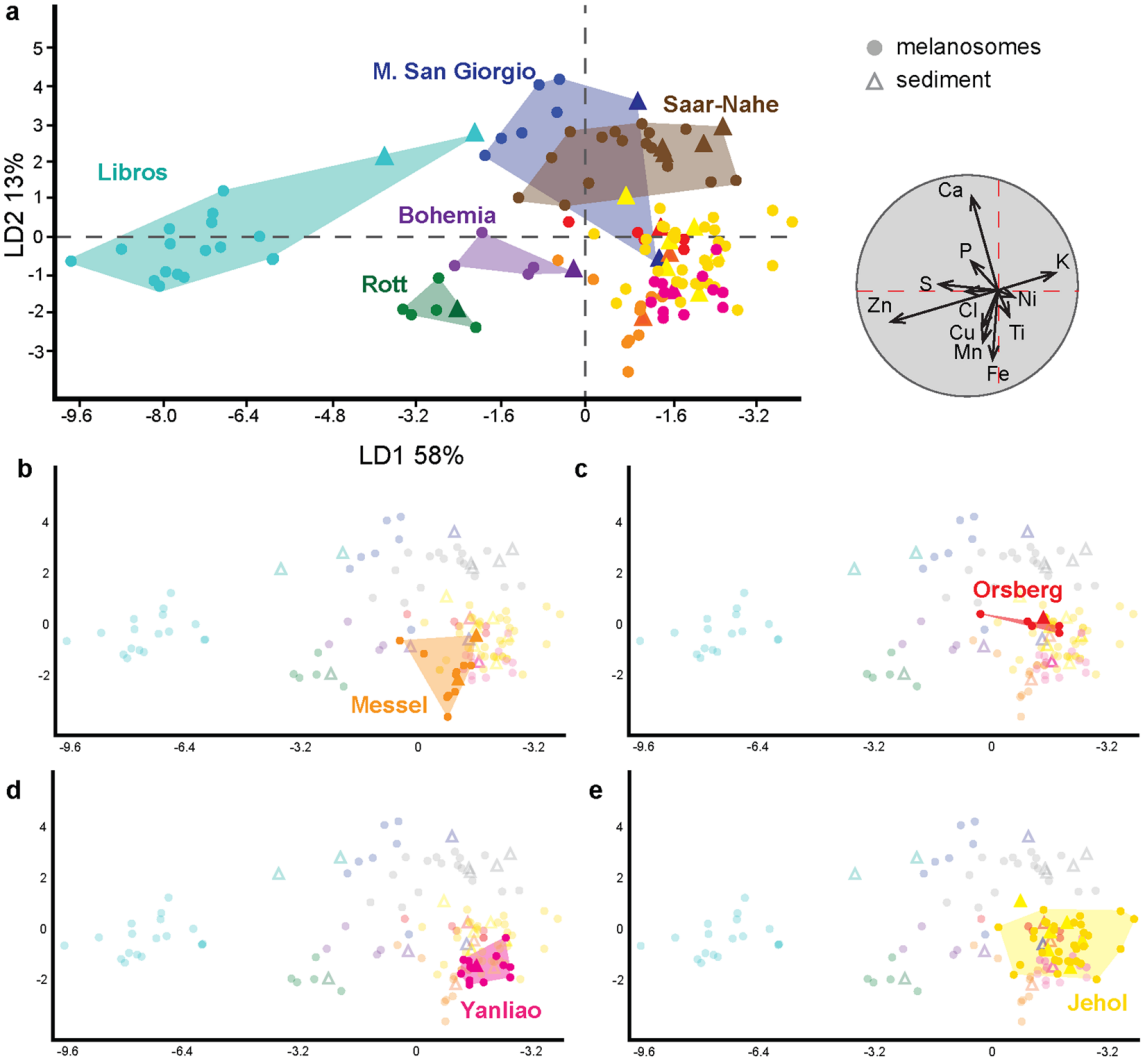
Linear Discriminant Analysis (LDA) of inorganic chemistry data for fossil melanosomes and sedimentary matrices. (**a**) Scatterplot of the LDA chemospace for the entire dataset with biplot (grey circle). Biplots show the most discriminating variables, i.e. those that contribute most to the separation among groups. (**b–e**) Panels highlighting the data for the Messel (**b**), Orsberg (**c**), Yanliao (**d**) and Jehol (**e**) biotas in the LDA plot in (**a**).

SRS-XRF maps reveal highly variable melanosome chemistry among fossil specimens (Supplementary Figs. S7–S19). Linear discriminant analysis (LDA) of the SRS-XRF data for the total dataset (n = 137, Fig. 2, Supplementary Data S1, S3) successfully resolves major trends in melanosome chemistry; the first and second linear discriminants (LD1 and LD2) combined explain 71% of the variation in the dataset (Fig. 2). The LDA plot shows that melanosome chemistry varies among fossil biotas (Fig. 2). This dominant inter-biota chemical signal primarily reflects differences in concentrations of Zn, Ca, Fe and K; other elements (in order of descending importance: S, Mn, Cu, P, Ti, Cl and Ni) vary less.

Broad trends in fossil melanosome chemistry in the dataset can be summarized as follows. Preserved melanosomes from Libros, Monte San Giorgio, Saar-Nahe, Rott and Bohemia are chemically distinct (Fig. 2). Those from Libros are enriched in Zn and S and plot in the left of the LDA chemospace. Melanosomes from Monte San Giorgio are enriched in Ca and P, whereas those from Saar-Nahe are enriched in Ca and K; collectively, these plot (with partial overlap) close to the centre of the chemospace. Melanosomes from Rott and Bohemia are enriched in Zn, Cu and Fe and plot in the lower left of the chemospace.

In contrast to these chemically distinct biotas, data for Messel, Orsberg, Yanliao and Jehol are clustered in the lower right of the chemospace. Subtle chemical differences are, however, apparent: melanosomes from Messel are relatively enriched in Fe, Mn and Ti (Fig. 2b), Orsberg melanosomes, in Ni and K (Fig. 2c), Yanliao melanosomes, in Fe, Ti and Ni (Fig. 2d), and Jehol melanosomes (Fig. 2e), in K, Ni, Ti and Fe. These chemical differences are better resolved when geological age is included as a variable in the analysis (Supplementary Fig. S20).

### Fossil melanosomes and the chemistry of the host sediment

The second major feature of the LDA plot in Fig. 2 is that biotas vary in the similarity between the inorganic chemistry of fossil melanosomes and that of their associated sedimentary matrix. Data for the latter usually plot within the chemospace for the associated fossil melanosomes; this applies to data from Saar-Nahe, Messel, Orsberg, Yanliao and Jehol (Fig. 2). For these biotas there is little to no difference between the chemistry of preserved melanosomes and that of the sediment. The exceptions to this trend are the data from Libros, Monte San Giorgio, Rott and Bohemia, where there are striking differences in the chemistry of soft tissues and the sediment. For Libros, melanosomes are enriched in Zn, and depleted in Ca and S, relative to the sedimentary matrix. For Monte San Giorgio, melanosomes are enriched in Ca, and depleted in K, Ni and Ti, relative to the host sediment. Lastly, melanosomes from Bohemia are enriched in Zn and Cu, and depleted in Mn and Fe, relative to the host sediment.

### Tissue-specific melanosome chemistry in fossil vertebrates

Detailed analysis of melanosome chemistry for individual fossil vertebrates reveals tissue-specific chemical signals in 14 specimens (Table 1, Supplementary Data S2, S3), including three described previously^17^ (see also Material and Methods).

**Figure 3:**
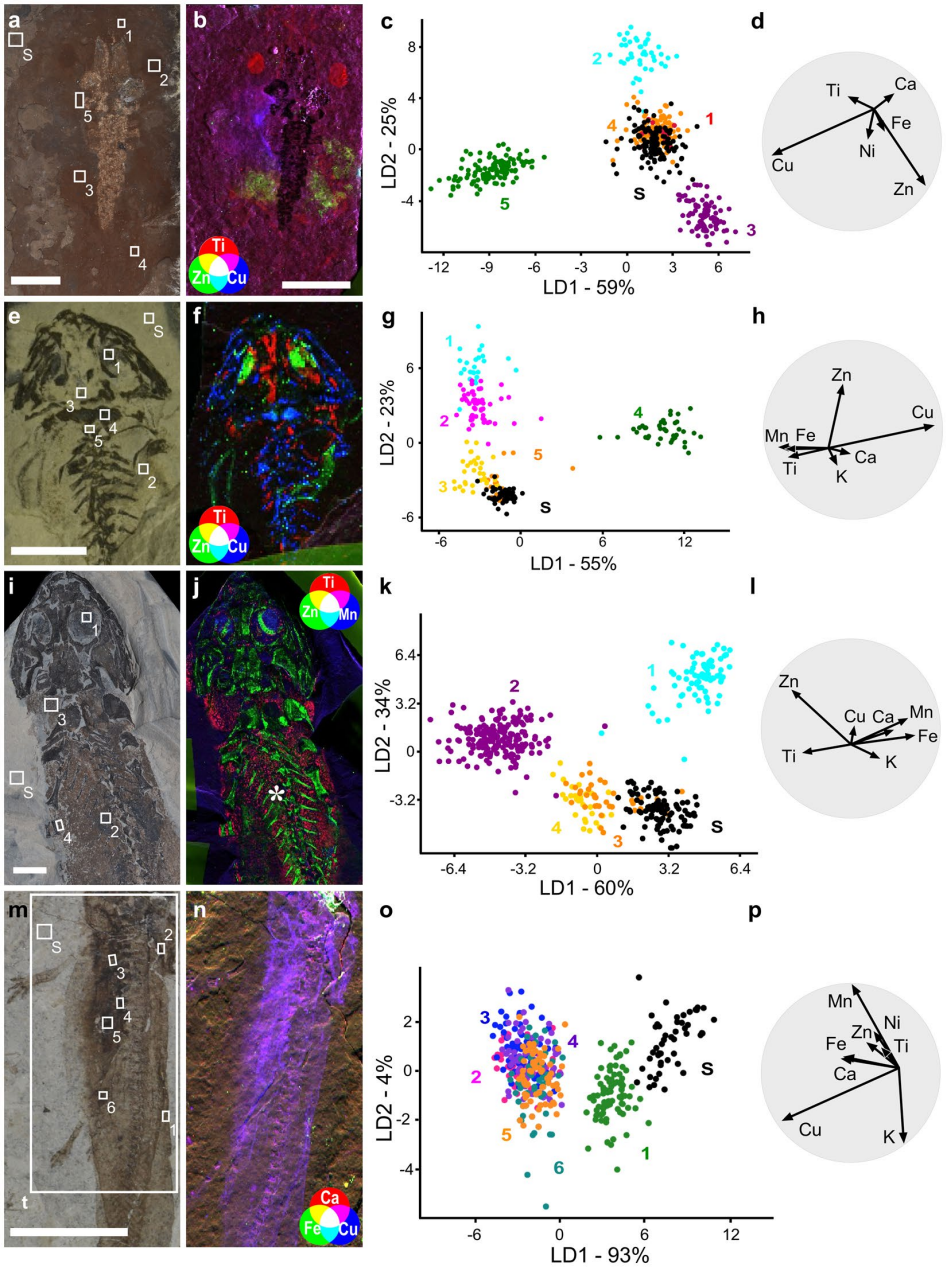
SRS**-**XRF and LDA analysis of individual fossil specimens. (**a–d**) *P. diluvianus* (NHML-30271); (**e–h**) *B. amplystomus* (NHMD-155208); (**i–l**) *M. credneri* (NHMB-MB-Am.-1187). (**m–p**); Amphibia indet. (CNU-SAL-NN2013002P); (**b,f,j,n**) SRS-XRF tricolor maps of each specimen; maps created using SMAK 1.50 https://www.sams-xrays.com/smak. Asterisks (*) denote internal tissues. (**c,g,k,o**) LDA plot for each specimen; colours and numbers denote different regions of interest; S, sediment. (**d,h,l,p**) biplots showing the most discriminating variables, i.e. those that contribute most to the separation among groups.

SRS-XRF maps reveal that melanosomes from the skin, eyespots, torso and/or abdomen are consistently associated with different elements (Fig. 3). Further, all melanosomes are usually chemically distinct from the host sediment associated with that fossil specimen. These features are reflected in the LDA plots (Fig. 3), which show a clear separation in chemospace between the host matrix, eyespot (when preserved) and one or more soft tissue regions in the torso/abdomen. The only exception is *Eopelobates* sp. from Orsberg, which shows no difference between melanosomes and the sedimentary matrix (see Supplementary Text). In some fossil specimens (see Supplementary Text), the inorganic chemistry of the skin melanosomes is more similar to that of the sediment than of other tissues; this phenomenon does not consistently apply to any particular species or vertebrate class in the dataset. Melanosomes from internal soft tissue regions are usually enriched in Zn and/or Cu (e.g., Fig. 2), but there is no consistent chemical signal for the eyespot, which can be enriched in Zn, Ti or Mn. The skin can be enriched in any or all of the following metals: Ca, Ti, Cu and Zn. Only specimens from Libros (see ref. ^17^), Rott, Saar-Nahe and one specimen from Monte San Giorgio show clear tissue-specific chemical differences in melanosomes. ANOVA and Tukey post hoc tests confirm that the chemical differences among all regions analysed are statistically significant for almost all elements for each fossil specimen (Supplementary Data S4).

The chemical data and anatomical interpretation for each specimen are presented in Supplementary Figs. S1–S19 and discussed in detail in the Supplementary Text.

### Metal enrichment in fossil amphibian melanosomes

**Figure 4:**
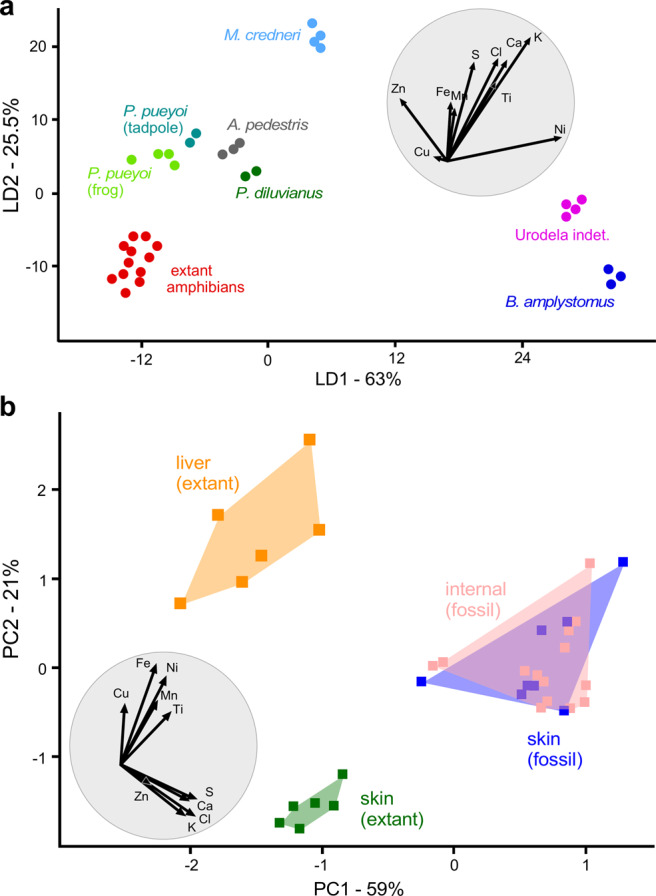
Multivariate analysis of the inorganic chemistry of modern and fossil melanosomes from amphibian tissues. (**a**) Linear Discriminant Analysis (LDA) of extant and fossil species. (**b**) Principal Component Analysis (PCA) of the inorganic chemistry of melanosomes from skin and liver. Grey circles are biplots. Note extensive overlap of the data for these two tissues in fossil amphibians only.

Comparison of the chemical data for extant and fossil representatives of the same vertebrate class is possible only for the amphibians in our dataset (Fig. 4a, Supplementary Data S5, S6), due to insufficient sample size for the other classes. Similarly, there are insufficient data for analysis of intra-taxonomic differences from each biota.

LDA analysis of the amphibian data shows that fossil melanosomes are enriched in all elements relative to modern melanosomes; the latter cluster in the lower left of the chemospace (Fig. 4a). PCA analysis of the chemical data reveals a tissue-specific signal: melanosomes from the liver and skin in extant amphibians are chemically distinct (described previously in ref. ^17^). In contrast, fossil melanosomes from the skin and internal organs (interpreted as liver melanosomes as these are the primary source of internal melanosomes in extant amphibians^16^) overlap extensively in chemospace (Fig. 4b).

## Discussion

Our data show clear differences in the inorganic chemistry of melanosomes from different biotas. These differences relate primarily to variation in the concentrations of the following elements (in descending order): Zn, Ca, Fe and K. Given that preserved melanosomes and sediment overlap in chemospace for most of the biotas studied (Rott, Orsberg, Messel, Jehol, Yanliao, Saar-Nahe), the observed broad-scale biota-level differences in melanosome chemistry almost certainly relate to broad differences in the chemistry of the host sediment among biotas. The primary controls on melanosome chemistry could therefore relate to chemical fingerprints that are generated *in vivo* as a result of environmental factors such as the chemistry of local waterbodies and vegetation, and thus ultimately the chemistry of the local bedrock. In addition / alternatively, major biota-level differences could reflect differences in the diagenetic histories of the host sediments. Chemical separation of the cluster of biotas in Fig. 2 when age is included in the analysis (Supplementary Fig. S20) suggests that time is also a control on the fidelity of the metal inventory of fossil melanosomes, whereby older melanosomes are more likely to be chemically altered. This does not, however, preclude preservation of components of original chemical signatures in older fossils: specimens from the Permian Saar-Nahe biota retain tissue-specific chemistry. The fidelity of preservation of melanosomes in fossil biotas should therefore be assessed on a case-by-case basis by chemical comparison of melanosomes and host sediment.

Accurate interpretation of trace metal concentrations in fossil melanosomes requires an understanding of how melanosomes from different tissues respond to elevated concentrations of elements in pore fluids under different pH / Eh conditions, and of the original elemental concentrations *in vivo*. Studies of the relationship between diet and melanisation have produced conflicting results (discussed in ref. ^36^ and references therein); in particular, whether the dietary intake of macro- and micronutrients affects the tissue-specific inorganic chemistry of melanosomes is uncertain. Hence, we consider diagenetic overprinting to be the most plausible explanation for the enrichment in elements observed in some fossil vertebrates.

In fossil vertebrates from Libros, Bohemia and Monte San Giorgio, preserved melanosomes are always separated in chemospace from their associated host sediment. These are also biotas for which melanosomes in most or all specimens exhibit tissue-specific chemical variation. Collectively, these data suggest that melanosomes that are more chemically distinct from the surrounding sediment are more likely to retain tissue-specific signals. Conversely, the clustering of the data for the other biotas suggests that melanosome chemistry converges during diagenesis, potentially due to common pathways for chemical transformation, especially metal enrichment, but this hypothesis should be tested experimentally.

Our data for melanosome-rich soft tissues from individual fossil specimens reveal broad trends in tissue chemistry relative to the host sediment: melanosomes are usually relatively enriched in Ca, Ti, Cu and Zn. The exception to this trend is the skin: in most specimens, skin melanosomes overlap in chemospace with the sediment. This may reflect the thin nature of the layer of skin melanosomes (typically *ca*. 3 µm, versus 11–25 µm for the layer of non-integumentary melanosomes^37^, Supplementary Fig. S21). Skin melanosomes may thus be intrinsically difficult to detect with SRS-XRF. In addition/alternatively, skin melanosomes may be predisposed to chemical alteration because of their close juxtaposition with the sedimentary matrix and the potential for chemical interactions with pore fluids during burial.

Many of the fossils analysed here reveal striking differences in inorganic chemistry among melanosomes from different body regions (see Supplementary Text). These data support our findings from a more limited fossil dataset^17^ that preserved melanosomes from individual fossil specimens can retain a tissue-specific inorganic chemical signature, allowing the discrimination of some internal soft tissues from the skin (see Supplementary Text). Despite this, the chemistry of melanosome-rich soft tissues in at least some fossils is unlikely to be wholly original. For example, melanosomes in the eyespots from the Saar-Nahe amphibians (*B. amplystomus*, *M. credneri* and *A. pedestris*) are often enriched in Ti or Mn; enrichment in these elements is not observed in melanosomes from any extant vertebrate taxon or tissue^17^. Similarly, fossilized melanosomes in the skin of fossil vertebrates are often enriched in Ti (see also ref. ^17^). This contrasts with skin melanosomes in extant vertebrates, which are usually associated with Ca and Zn^17^. Although it is conceivable that early-branching or basal representatives of various taxonomic groups may differ markedly in melanosome inorganic chemistry to extant analogues, the conserved nature of these chemical signals in extant taxa^17^ suggests that fossil members of the relevant crown groups should show a similar chemistry to extant forms. The differences in melanosome chemistry between fossil and extant vertebrates observed here are therefore likely to reflect differences in diagenetic history, in particular, exposure to pore fluids of different chemistry. Hence, fossil melanosomes with elevated concentrations of metals that are not associated with the relevant tissues in extant analogues are unlikely to preserve a real biological signal; the preserved chemistry is most plausibly interpreted as diagenetically altered, whereby concentrations of certain metals increase (and, potentially, concentrations of others may decrease) during diagenesis. Hence, the enrichment of Ti and Mn in the fossil melanosomes analysed here suggests that the chemistry of the latter has been altered during diagenesis. Interpretation of other chemical signals, however, is less straightforward. The fossil amphibian melanosomes show enrichment (by up to two orders of magnitude) of other elements, all of which are known to associate with melanosomes *in vivo*. Taphonomic experiments could provide useful data on the extent of enrichment possible (without additions from external sources) during decay and diagenesis.

Critically, the absence of a pervasive and consistent tissue-specific chemical signal in our total dataset of fossil amphibians supports our hypothesis that extrinsic processes can modify concentrations of elements associated with fossil melanosomes, even though the latter can retain a tissue-specific signal at the level of individual specimens.

The evidence presented here for the diagenetic incorporation of metals (e.g., Cu and Ti) into melanosomes has implications for the use of trace metal chemistry to infer original melanin-based integumentary colour in ancient organisms^8,10^. Cu has been proposed as a biomarker for eumelanin^8^ and Zn, for phaeomelanin^10^. Highly elevated concentrations of these metals in fossil melanosomes relative to extant examples would call inferences of colour into question, providing diagenetic addition of metals cannot be excluded. Concentration of these elements in fossil melanosomes is more likely original where it is consistent with other chemical data that support a melanic origin, e.g. elevated concentrations of melanin monomers (e.g., benzothiazole^10^) and/or derivatives^38^, or suites of other diagnostic molecular fragments^20^. Further, taphonomic experiments are required to test whether eumelanin can incorporate Zn, and phaeomelanin Cu, during diagenesis which might further hamper interpretations of original chemistry.

Metal binding capacity (the ability of a molecule to bind certain elements) and affinity (the strength of the bond) are well characterized for both synthetic and natural melanins^31,33^. Melanin can chelate metals from (and release metals to) the environment under various conditions of pH and metal availability^33^. This can occur without loss of molecular integrity (albeit with minor to major changes in the physical properties of the molecule^1,33^). In *Sepia* melanin, Mg, Ca and Zn bind exclusively to carboxylic acid groups (COOH), Cu binds primarily to hydroxyl (OH) groups^33,39–41^ and Fe binds exclusively to OH or amine groups (NH)^41^. Biological associations between melanin and metals present *in vivo* can, however, be altered by changes in local pH and metal concentrations^39,41^. Further, whether the above binding relationships apply to all natural melanins, including those in vertebrates, is unknown. In our study, Ca and Fe are usually relatively enriched in the sediment (up to *ca*. 2500 µm/cm^2^ for Ca and up to *ca*. 3500 µm/cm^2^ for Fe) and rarely enriched in melanosomes from the skin and internal organs; instead, the latter tissues are usually enriched in Zn, Cu and Ti. Assuming the binding relationships above are broadly similar among invertebrate and vertebrate melanins, the elemental distributions in the fossil amphibians could reflect a decrease in ambient pH during diagenesis, promoting the chelation of Zn, Cu and Fe to melanosomes, rather than Ca^39,41^. This hypothesis is supported by experimental evidence that exposure of melanin to a concentrated metal solution can result in binding of metal ions to all functional groups available^39^. Unlike the sedimentary matrix, the fossil melanosomes are rarely enriched in Fe, but this is not unexpected: binding rates of Cu to melanin are higher than those for Fe^39^.

The capacity of melanin to chelate metals is controlled by its molecular structure. Melanin, especially eumelanin, comprises subunits of 5,6-dihydroxyindole (DHI) and 5,6-dihydroxyindole-2-carboxylic acid (DHICA). Fossil melanin, however, is diagenetically altered^22,30^: it contains a higher ratio of PTeCA/PTCA relative to melanin from extant vertebrates^22^. This has been attributed to cross-linking among DHI and DHICA units due to thermal maturation during burial^22^. Previous experiments^29^ have shown that during thermal maturation, part of the DHICA unit of eumelanin undergoes decarboxylization, yielding additional DHI. Thermal maturation therefore induces reconfiguration of the constituent monomers of melanin. The impact of this molecular reconfiguration on the metal content and binding capacity of melanin has not been explored fully. It is, however, likely that the loss of the carboxylic group (COOH) in DHICA and the generation of additional, diagenetic, DHI units would provide additional hydroxyl (OH) groups that preferentially bind Cu and Ti^31^; this is consistent with the elevated concentrations of these elements in the fossils.

Decay typically results in pH fluctuations in and around a carcass^42–45^, resulting in a release of cations that, if pH conditions are favourable^33^, could potentially bind to melanin. Subsequent diagenesis may involve changes in temperature, pressure, pH and pore fluid metal concentrations. Both these extrinsic environmental variables and the intrinsic chemical properties of melanosomes (DHICA-DHI ratio, binding capacity and binding affinity) are likely to be major controls on the preserved chemistry of melanosomes in fossils.

In conclusion, our study reveals the major controls on the chemistry of fossil melanosomes. The diagenetic history of the fossil-bearing sediments, especially the pore fluid chemistry, controls variation in melanosome chemistry at the biota-level; this is superimposed by a second, biological, control: that of tissue type, whereby individual tissues vary in melanosome chemistry. The chemistry of the depositional environment, i.e., water chemistry and/or diet (ultimately relating to the nature of the local bedrock) may also influence melanosome chemistry but this requires additional testing as the existing literature is inconclusive. Although discrimination of different soft tissues using melanosome chemistry is possible for fossils from some biotas, e.g., Libros, Rott and Saar-Nahe, the chemistry of fossil melanosomes cannot be considered fully original even in these fossils. Contra claims that SRS-XRF is of limited value in fossil melanin research^46^, the ability to characterise the chemistry of an entire fossil plus its associated sedimentary matrix is clearly fundamental to resolution of important trends in melanosome preservation both within and between specimens. Further, the approach used here provides a novel and essential mechanism for discriminating between more and less diagenetically altered fossil melanins in a rapid, non-destructive way. Future experimental studies will characterise the response of the elemental chemistry of natural melanins from different tissues to different diagenetic conditions, thus providing empirical data to aid attempts to discriminate biological from diagenetic components of the melanosome chemical signal.

## Material and Methods

### Fossil specimens

Fossil specimens are from the following institutions: Hessisches Landesmuseum Darmstadt (HLMD), Institute of Vertebrate Palaeontology and Palaeoanthropology Beijing (IVPP), Museum für Naturkunde Berlin (MFNB), National Museum of Denmark (NHMD), Natural History Museum London (NHML), Paläontologisches Institut und Museum, Universität Zürich (PIMUZ), and Capital Normal University, Beijing (CNU). Specimens include amphibians, reptiles and birds, and represent different ages and depositional environments (Table 1). The data for the Libros specimens and for one bat from Messel are from ref. ^17^. The data for fossil vertebrates from the Jehol biota are described in detail in McNamara *et al*., *in prep*. SRS-XRF data for individual fossil specimens from Rott, Bohemia, Messel, Yanliao, Monte San Giorgio and Saar-Nahe are treated in detail herein.

### Scanning electron microscopy

Samples of fossil soft tissues were dissected using sterile scalpels and placed on aluminium stubs, coated with Au/Pd and examined using a JEOL IT100 VP-SEM at an accelerating voltage of 20 kV and working distance of 8–10 mm.

### Synchrotron rapid scanning-X-ray fluorescence (SRS-XRF)

Synchrotron rapid scan-X-ray fluorescence data were collected at the Stanford Synchrotron Radiation Lightsource using beam line 10-2. The incident X-ray energy was set to 11 keV using a Si (111) double crystal monochromator with the storage ring containing 500 mA in top off mode at 3.0 GeV. A focused beam of 25 × 25 μm was provided by using a tungsten aperture. The incident X-ray intensity was measured with a nitrogen-filled ion chamber. Samples of fossil soft tissues (*Neusticosaurus edwardsii*, PIMUZ-T3749) and fossil-bearing slabs (all other specimens) were mounted at 45° to the incident X-ray beam and were spatially rastered at 50 ms / pixel dwell time with a pixel size of 25 μm x 25 μm. The entire fluorescence spectrum was collected at each data point and the intensity of fluorescence lines for selected elements (P, S, Cl, K, Ca, Ti, Mn, Fe, Ni, Cu and Zn) was monitored using a silicon drift Vortex detector; these elements are usually present in our samples between 0.1 and 3600 µg/cm^2^. Fluorescence intensities were corrected and normalized for detector deadtime and variation in I0 to allow comparison among samples. The concentrations of each element in µg/cm^2^ were calibrated using NIST traceable thin film elemental standards. Data processing was performed using MicroAnalysis Toolkit software^47^. Regions of interest for quantitative analysis were selected from soft tissues and sedimentary matrix for each fossil specimen. For each region of interest, raw pixel data was extracted and mean and standard deviation values calculated for the concentrations of each element (Supplementary Datas S1 and S2).

### Peak interpretation

XRF spectra were produced for several regions of interest (Supplementary Figs. S22–S27) using the MCA fitting spectra function in MicroAnalysis Toolkit, which uses PyMCA algorithms^48^. The MCA spectra show well-constrained k-peaks for all elements of interest. All peaks were fitted using spectrometer zero, spectrometer gain, and detector width in order to achieve the best fit for the spectrum of interest in the MicroAnalysis Toolkit (Supplementary Data S7).

### Statistical analysis

The differences in elemental concentration among biotas were assessed using LDA in PAST. Differences in elemental concentrations among regions of interest (including melanosomes and sediment) within and among taxa were assessed using Linear Discriminant Analysis (LDA) and ANOVA in PAST^49^. Principal Components Analysis (PCA) was used to assess differences in the trace element chemistry between skin and liver in extant and fossil amphibians. It was not possible to analyse these data using LDA because the latter requires a minimum of three groups to produce a chemospace, whereas only two groups (i.e., tissues) are considered here.

## Supplementary information

Supplementary Data S1.

Supplementary Data S2.

Supplementary Data S3.

Supplementary Data S4.

Supplementary Data S5.

Supplementary Data S6.

Supplementary Data S7.

Supplementary information, figures and data legends.
