# Supplementary material for: Hierarchical biota-level and taxonomic controls on the chemistry of fossil melanosomes revealed using synchrotron X-ray fluorescence
Source: Sci Rep. 2020 Jun 2;10:8970. doi: 10.1038/s41598-020-65868-3 (PMC7265528; doi:10.1038/s41598-020-65868-3)
Supplement: Supplementary file 8 — Supplementary information, figures and data legends. [file 41598_2020_65868_MOESM8_ESM.docx]

**Supplementary Information**

**Hierarchical biota-level and taxonomic controls on the chemistry of fossil melanosomes revealed using synchrotron X-ray fluorescence**

Valentina Rossi^1^*, Sam Webb^2^, Maria McNamara^1*^

*^1^School of Biological, Earth and Environmental Sciences, University College Cork, North Mall, Cork T23 TK30, Ireland*

*^2^Stanford Synchrotron Radiation Lightsource (SSRL), SLAC National Accelerator Laboratory, Menlo Park, CA 94025, USA*

*Corresponding authors: [valentina.rossi@ucc.ie](mailto:valentina.rossi@ucc.ie); [maria.mcnamara@ucc.ie](mailto:maria.mcnamara@ucc.ie);

**Supplementary text**

**Supplementary figures: S1–S27**

**Supplementary Data legend: S1–S7**

**References**

**Supplementary text**

The text that follows comprises a detailed description of SRS-XRF, LDA and ANOVA results for each fossil specimen analysed in this study.

**Taxon-specific trace element chemistry.** The SRS-XRF tricolour map and LDA plot of the fossil tadpole *Palaeobatrachus diluvianus* from Rott (NHML-30271, Figs. 3a–d, Supplementary Data 1–3) show that Cu is enriched in a region to the posterior and left of the cranium that corresponds to the position of the liver in extant tadpoles^1^. Zn is enriched in two paired regions in the abdomen that could correspond to the kidneys^1^. Ti is enriched in the eyespots and, to a lesser extent, the skin. The data for the skin and sedimentary matrix overlap extensively in the LDA chemospace (Fig. 3c and d). Despite this, ANOVA and Tukey post hoc tests confirm that the differences in element concentrations among all regions analysed are statistically significant for almost all elements (Supplementary Data S4).

These patterns of metal enrichment differ to those in the temnospondyl *Brachierpeton amplystomus* from Saar-Nahe (NHMD-155208, Figs. 3e–f, Supplementary Data 1–3). Zn is enriched in the eyespots and the external gills and Ti, in the skin. Cu is enriched in a thin, fibrous organic layer that lacks melanosomes and occurs locally on the bones (see Supplementary Fig. S1). In the LDA chemospace, all body regions and the sediment plot separately, except for minor overlap between the sediment and skin (i.e. region of interest (ROI 3) and possible regions of internal soft tissues (ROI 5) (Fig 3g and h) and between the eyespot and the gills. ANOVA and Tukey post hoc tests confirm that the chemical differences among all regions analysed are statistically significant for almost all elements (Supplementary Data S4).

In *Micromelerpeton credneri* from Saar-Nahe (NHMB-MB-Am.-1187, Figs. 3i–l, Supplementary Data 1–3), Ti is relatively enriched in the skin; Zn is enriched in soft tissue regions adjacent to the vertebral column (asterisk in Fig. 3j). The eyespots are enriched in Mn, Fe and, to a lesser extent, Ca. All body regions and sediment plot separately in the chemospace, excepting minor overlap between the data for the skin (ROIs 3 and 4) and sediment (Fig. 3k and l). ANOVA and Tukey post hoc tests confirm that the differences among all regions analysed are statistically significant for almost all elements (Supplementary Data S4).

All of the soft tissues of the fossil salamander from Yanliao (CNU-SAL-NN2013002P, Fig. 3m–p, Supplementary Data 1–3) are enriched in Cu and, to a lesser extent, Ca. The map for Cu shows subtle variations in concentration among tissue regions, but the latter are not sufficiently well defined to interpret internal anatomy. The melanosome-rich tissues are not enriched in any other elements relative to the sedimentary matrix. The data for soft tissue regions ROIs 2–4 overlap extensively in the LDA chemospace (Fig. 3o and p); skin and sediment plot separate to each other and to other regions of interest. ANOVA and Tukey post hoc tests confirm that the differences among all regions (soft tissues and sediment) analysed are statistically significant for almost all elements, but differences among some pairs of tissues and tissue-sediment pairs are not significant (Supplementary Data S4).

In the frog *Palaeobatrachus luedecki* from Bohemia (NHML-OR35814, Supplementary Figs. S2, S13, Supplementary Data 1–3), Zn is enriched in the eyespots. Ca, Zn, and to a lesser extent, K and S are enriched in a region in the torso that may correspond to internal organs (ROI 2). The chemistry of each soft tissue is distinct from the others and from the sedimentary matrix (Supplementary Fig. S1d). ANOVA and Tukey post hoc tests confirm that the differences among all regions analysed are statistically significant for almost all elements (Supplementary Data S4).

SRS-XRF maps of *Eopelobates sp*. from Orsberg (NHMB-Am908, Supplementary Figs. S3, S14, Supplementary Data 1–3) do not discriminate between melanosome-rich soft tissues and the sediment. This is also evident in the LDA plot, which shows extensive overlap among all of the soft tissue ROIs and the sedimentary matrix (Supplementary Fig. S3b). Except for P and S, the ANOVA test confirms that the differences in element concentrations are statistically significant. The Tukey post hoc test, however, shows that the differences in chemistry between many pairs of tissues or tissue-sediment pairs are not statistically significant (see details in Supplementary Data S4).

As in the fossil salamander from Yanliao (CNU-SAL-NN2013002P), Cu is the only element enriched in the soft tissues in the reptile CNU-VER-LB2009001 (Squamata indet.) from the same biota (Supplementary Figs. S4, S15, Supplementary Data 1–3). There is minor overlap between the data for the skin (ROI 1) and the second, unidentified, soft tissue region (ROI 2; this likely corresponds to organs in the abdominal cavity). ANOVA and Tukey post hoc tests confirm that the differences between both regions analysed are statistically significant for almost all elements (Supplementary Data S4).

In *Neusticosaurus edwardsii* from Monte San Giorgio (PIMUZ-T3749, Supplementary Figs. S5, S16, Supplementary Data 1–3), the skin (ROI 1) and a soft tissue region in the abdomen (ROI 3) show similar concentrations of Ca; a soft tissue region in the upper torso (ROI 2) is enriched in Mn, Cu, and also Ti. There is no overlap between the data for the skin, ROI 3 and the sediment in the LDA chemospace, but there is minor overlap between that for ROI 2 and the sediment. ANOVA and Tukey post hoc tests confirm that the differences among all regions analysed are statistically significant for almost all elements (Supplementary Data S4).

In *Neusticosaurus peyeri* from Monte San Giorgio (PIMUZ-T3412*,* Supplementary Figs. S6, S17, Supplementary Data 1–3), Ti is enriched in the eyespot, and Mn, S, Zn and Cu are enriched in other soft tissue regions (ROIs 2–4). There is no overlap in the LDA chemospace between any of the soft tissue regions and the sediment, but ROIs 2, 3 and 4 overlap extensively with each other. ANOVA and Tukey post hoc tests confirm that the differences among all regions analysed are statistically significant for almost all elements (Supplementary Data S4)

In *Apateon pedestris* from Saar-Nahe (NHMB-MB-Am 1220*,* Supplementary Figs. S7, S18, Supplementary Data 1–3), Cu and Zn are enriched in the skin (ROI4) and, especially, internal regions (ROIs 2 and 3); Cu and Ti are enriched in the eyespot. There is minor overlap between the eyespot, the other soft tissues and the sediment. The skin and ROIs 2 and 3 overlap extensively in chemospace, but not with the sediment. ANOVA and Tukey post hoc tests confirm that the differences among all regions analysed are statistically significant for almost all elements (Supplementary Data S4).

The melanosomes in the bird (Aves indet., HLMD-Me 5472) from Messel (Supplementary Figs. S8, S19, Supplementary Data 1–3) show enrichment in Cu and Zn, with only minor overlap with the sedimentary matrix in the LDA chemospace. ANOVA and Tukey post hoc tests confirm that the differences among all regions analysed are statistically significant for almost all elements (Supplementary Data S4).

**Supplementary Figures**


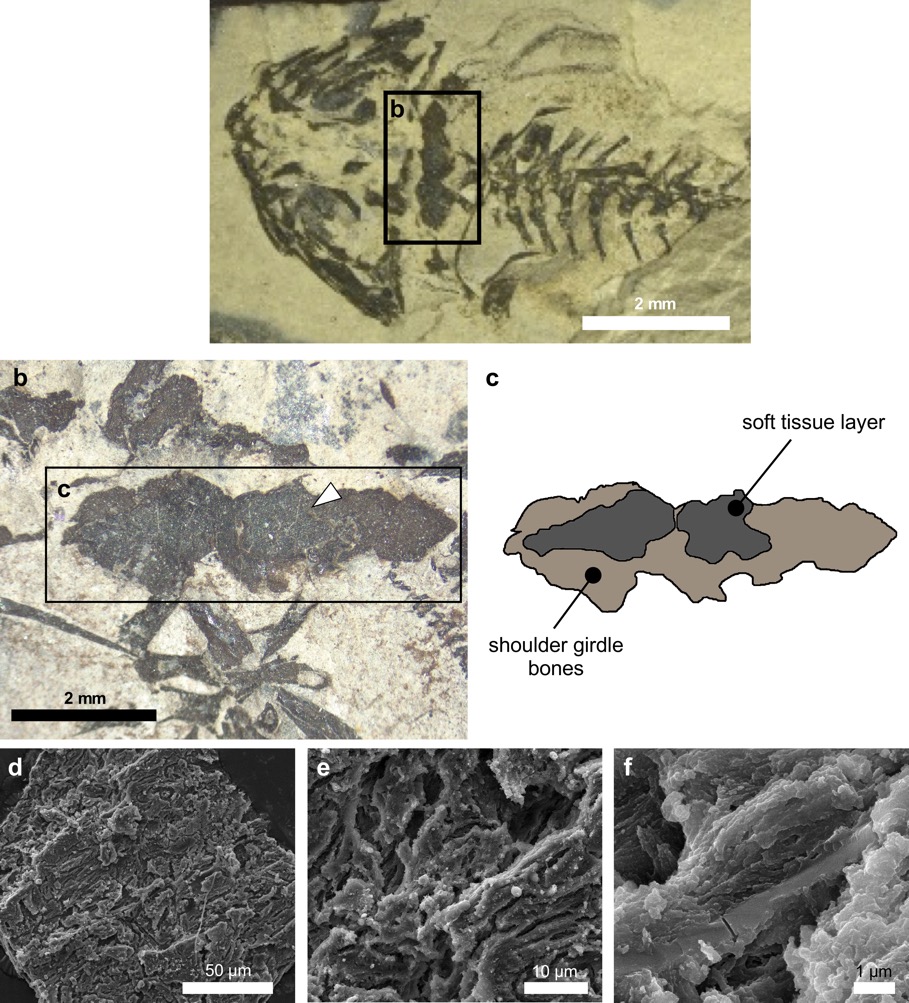


**Fig. S1** Details of a soft tissue layer in *B. amplystoma* associated with the bones of the shoulder girdle. (**a)** *B. amplystoma,* detail of the position of body region shown in **b**. (**b**) Magnified detail of the shoulder girdle bones; white triangle denotes sampling locations for SEM analysis. **c** Schematic illustration of the position of the soft tissue layer on the shoulder girdle bones. (**d**─**f**) Scanning electron micrographs of the soft tissue showing fibrous texture.

**
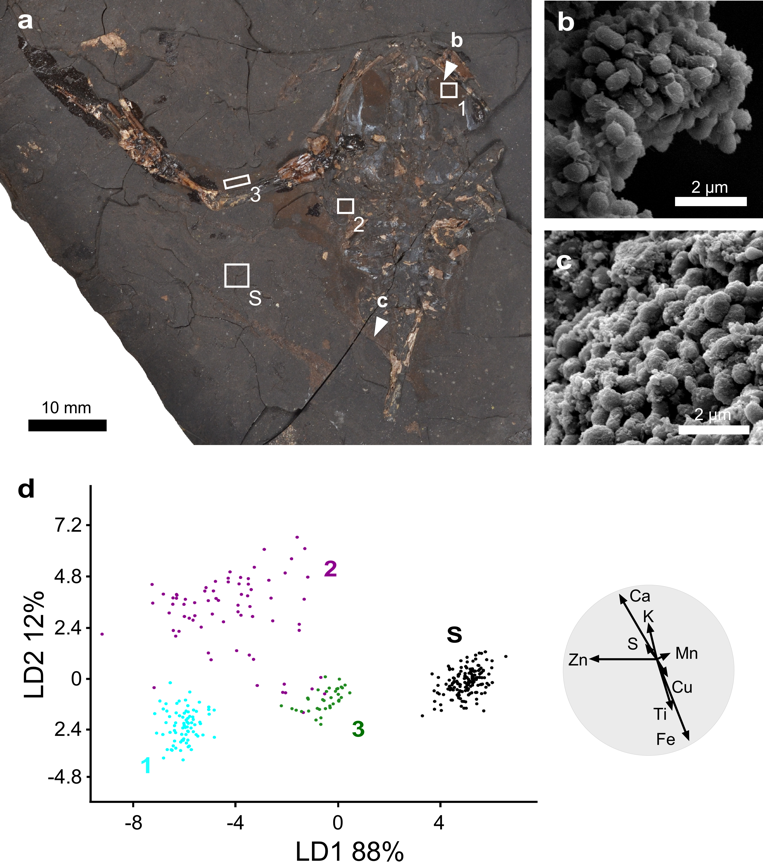
**

**Fig S2** *Palaeobatrachus luedecki* (NHML-OR35814). (**a**) White triangles denote sampling locations for SEM analysis; white rectangles and associated numerals denote regions of interest for LDA. S denotes sediment. (**b, c**) Scanning electron micrographs of melanosomes. (**d**) LDA plot and biplot (grey circle).

**
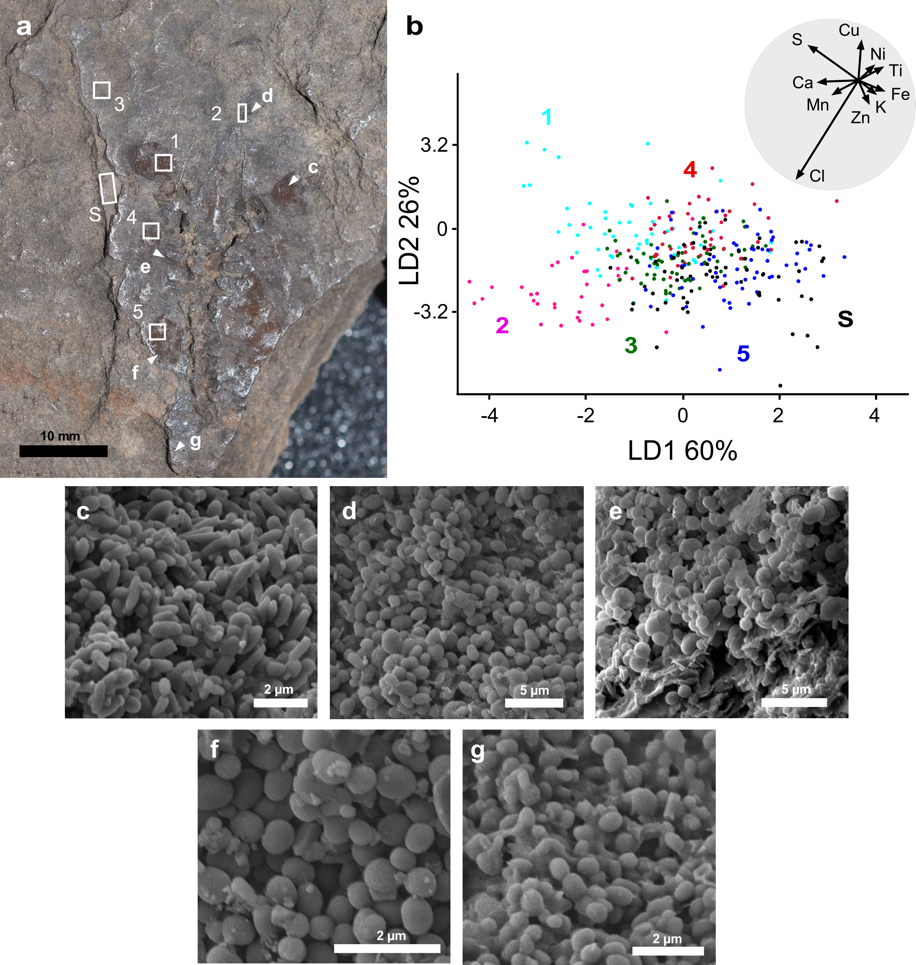
**

**Fig. S3** *Eopelobates sp.* (NHMB-Am908). (**a**) White triangles denote sampling locations for SEM analysis; white rectangles and associated numerals denote regions of interest for LDA. S denotes sediment. (**b**) LDA plot and biplot (grey circle). (**c**─**g**) Scanning electron micrographs of melanosomes.

**
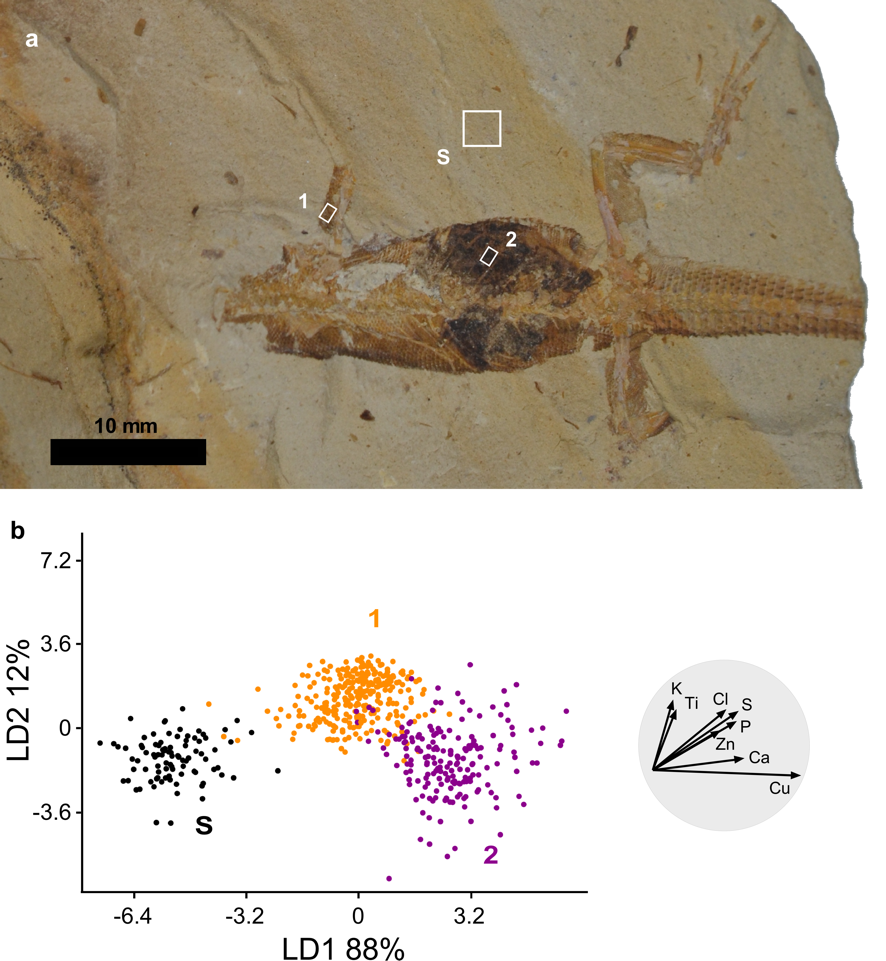
**

**Fig. S4** Squamata indet. (CNU-VER-LB2009001). (**a**) White rectangles and associated numerals denote regions of interest for LDA. S denotes sediment. (**b**) LDA plot and biplot (grey circle).


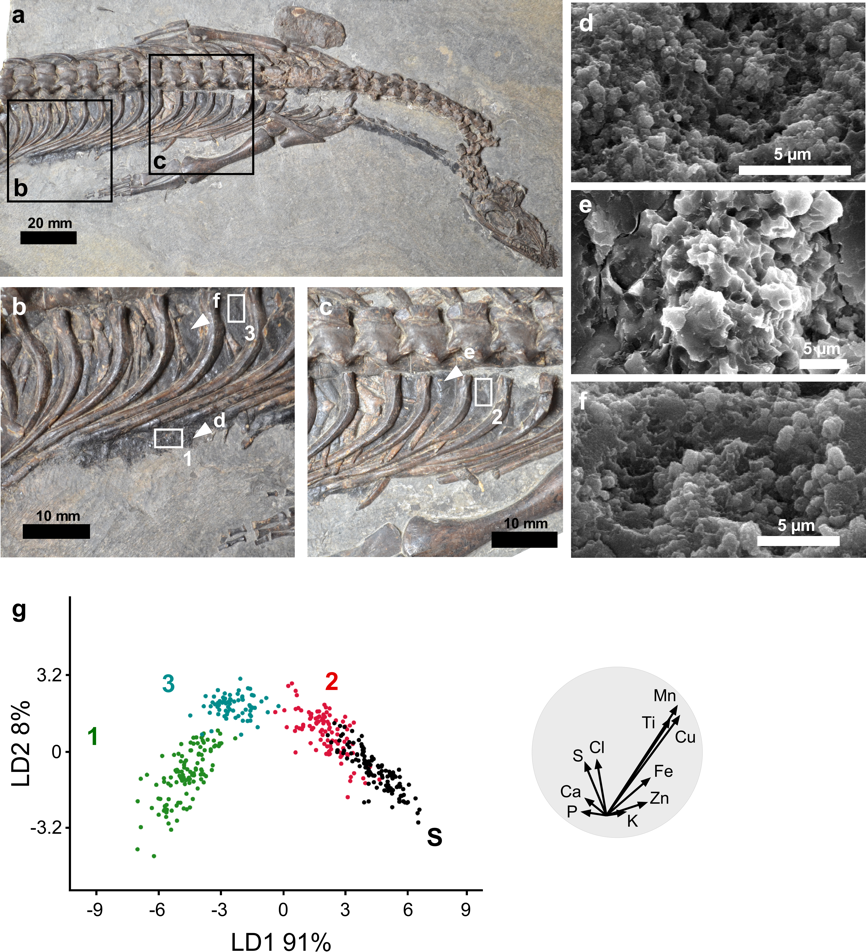


**Fig. S5** *Neusticosaurus edwardsii* (PIMUZ-T3749). (**a**) Photograph of the specimen. (**b, c**) Details of soft tissues: white triangles denote sampling locations for SEM analysis; white rectangles and associated numerals denote regions of interest for LDA. S denotes sediment. (**d**─**f**) Scanning electron micrographs of melanosomes. (**g**) LDA plot and biplot (grey circle).

**
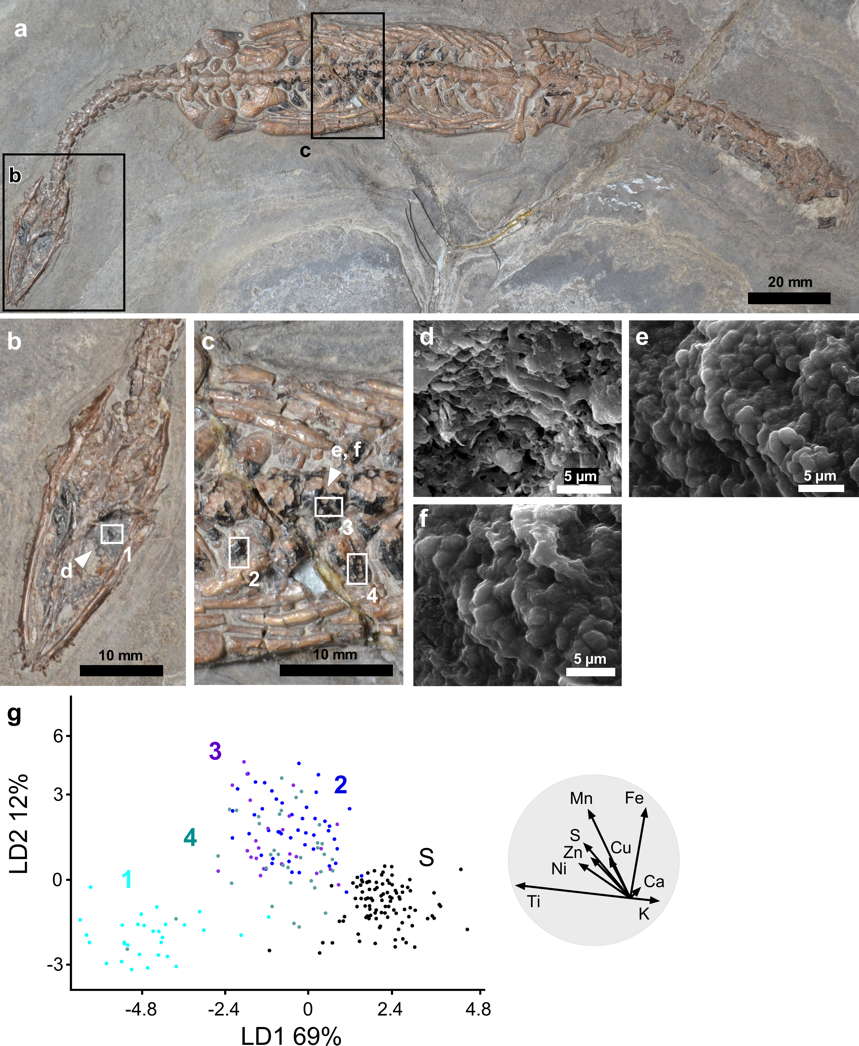
**

**Fig. S6** *Neusticosaurus peyeri* (PIMUZ-T3412). (**a**) Photograph of the specimen. (**b,c**) Details of soft tissues: white triangles denote sampling locations for SEM analysis; white rectangles and associated numerals denote regions of interest for LDA. S denotes sediment. Scale bars 10 mm. (**d**─**f**) Scanning electron micrographs of melanosomes. (**g**) LDA plot and biplot (grey circle).

**
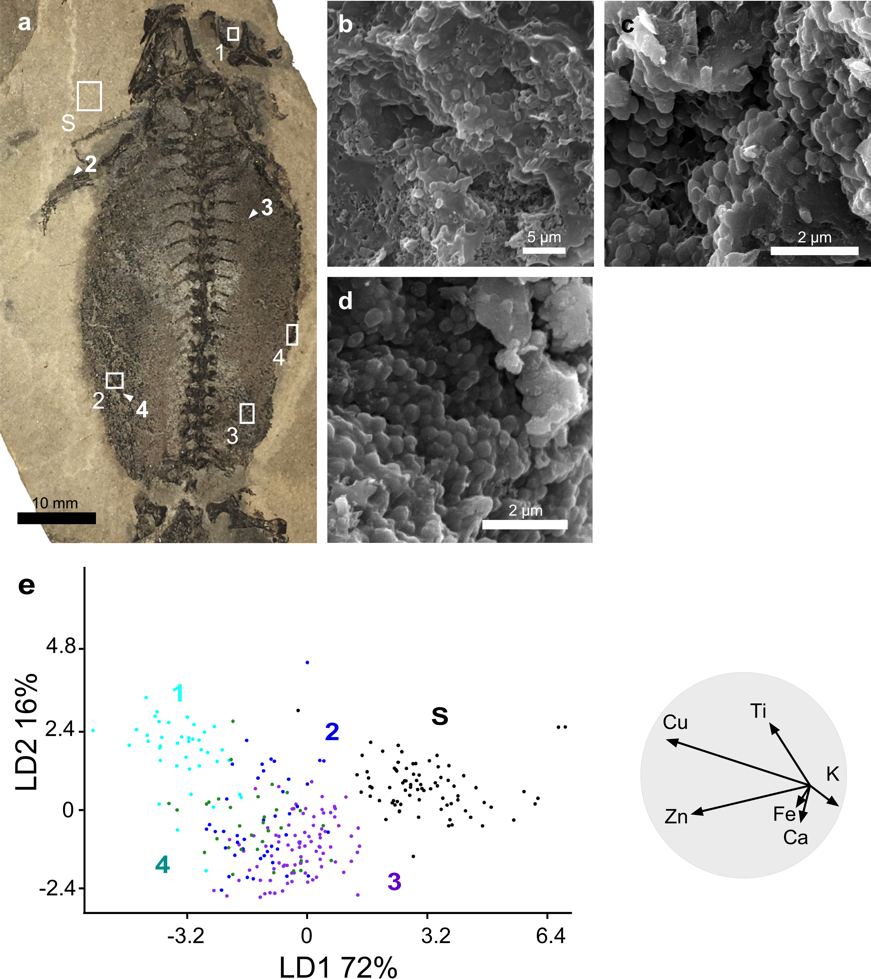
**

**Fig. S7** *Apateon pedestris* (NHMB-MB-Am 1220). (**a**) Photograph of the specimen. White triangles denote sampling locations for SEM analysis; white rectangles and associated numerals denote regions of interest for LDA. S denotes sediment. (**b**─**d**) Scanning electron micrographs of melanosomes. (**e**) LDA plot and biplot (grey circle).

**
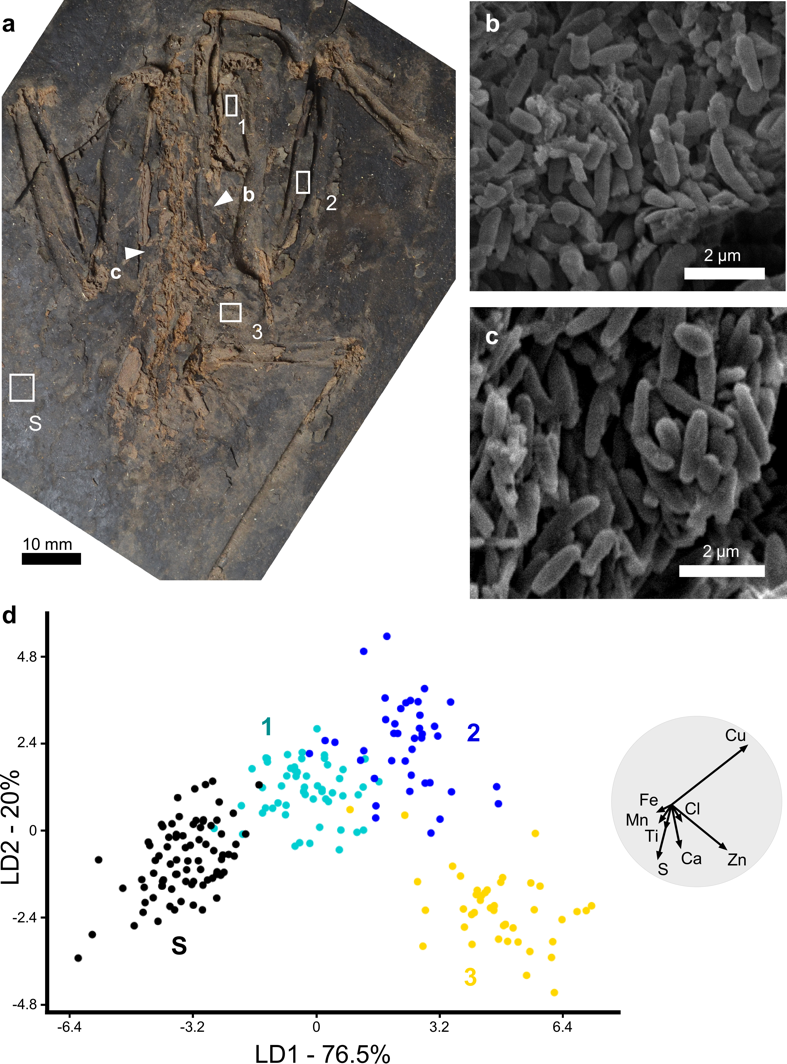
**

**Fig. S8** Aves indet. (HLMD-Me5472). (**a**) Photograph of the specimen. White triangles denote sampling locations for SEM analysis; white rectangles and associated numerals denote regions of interest for LDA. S denotes sediment. (**b, c**) Scanning electron micrographs of melanosomes. (**d**) LDA plot and biplot (grey circle).


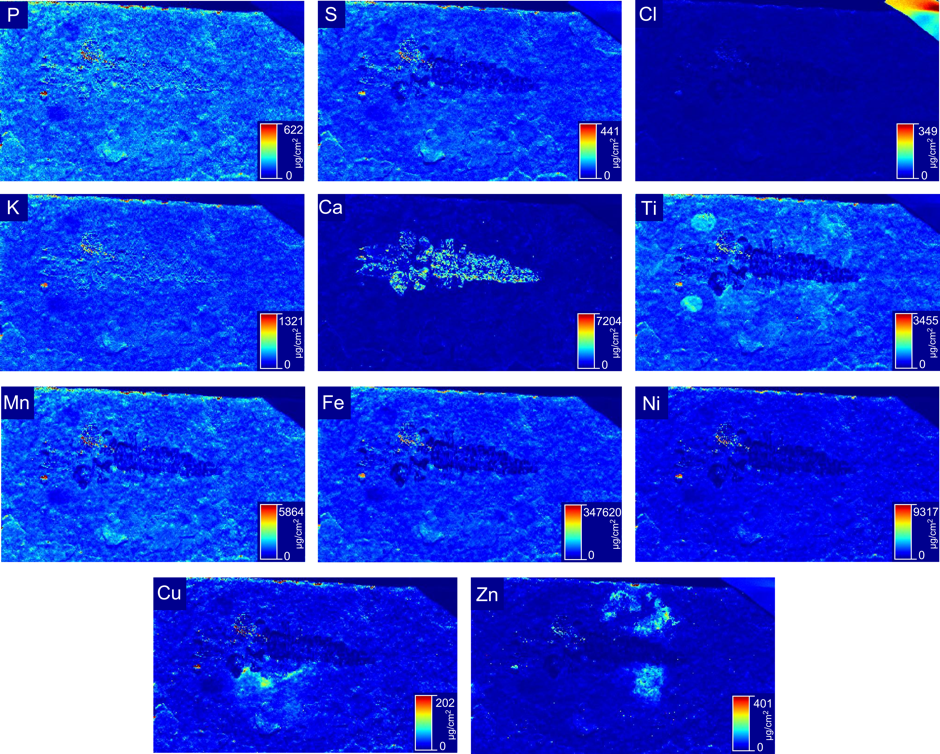


**Fig. S9** SRS**-**XRF maps of *Palaeobatrachus diluvianus* (NHML-30271) from Rott. Maps created using SMAK 1.50 <https://www.sams-xrays.com/smak>.


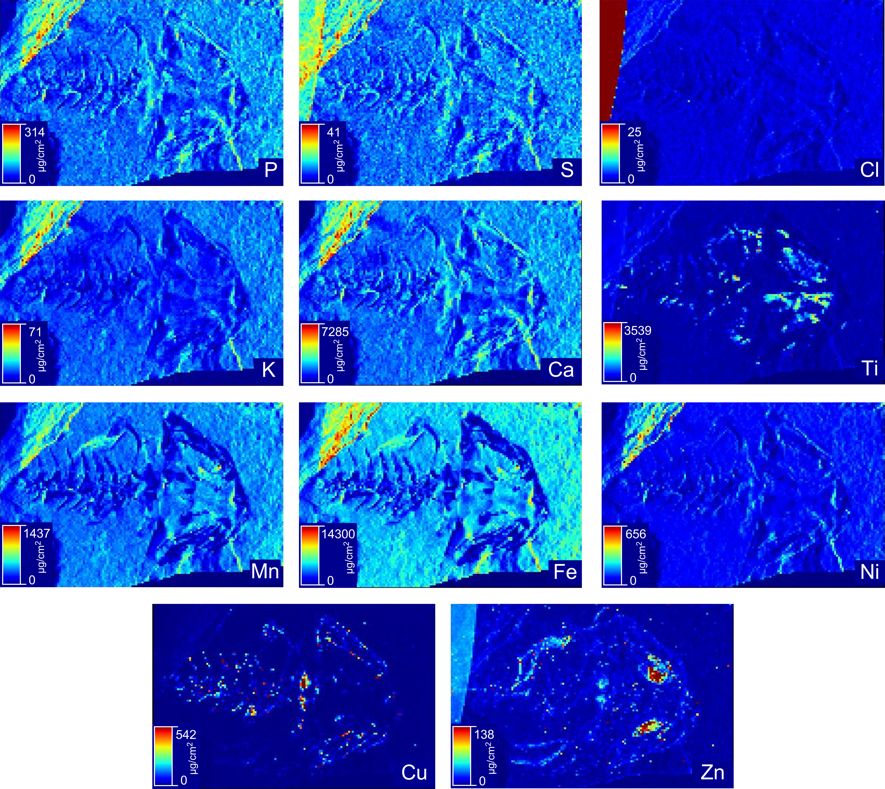


**Fig. S10** SRS**-**XRF maps of *Brachierpeton amplystoma* (NHMD-155208) from Saar-Nahe. Maps created using SMAK 1.50 <https://www.sams-xrays.com/smak>.


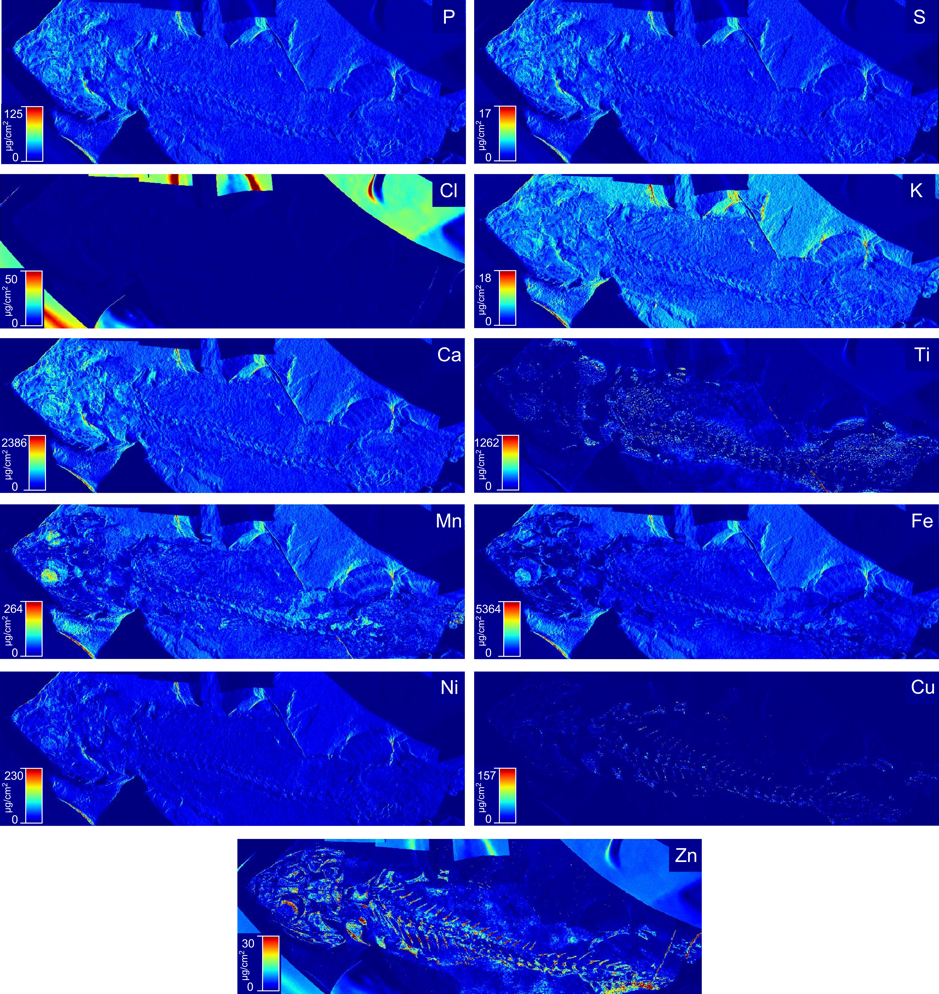


**Fig. S11** SRS**-**XRF maps of *Micromelerpeton credneri* (NHMB-MB-Am.-1187) from Saar-Nahe. Maps created using SMAK 1.50 <https://www.sams-xrays.com/smak>.


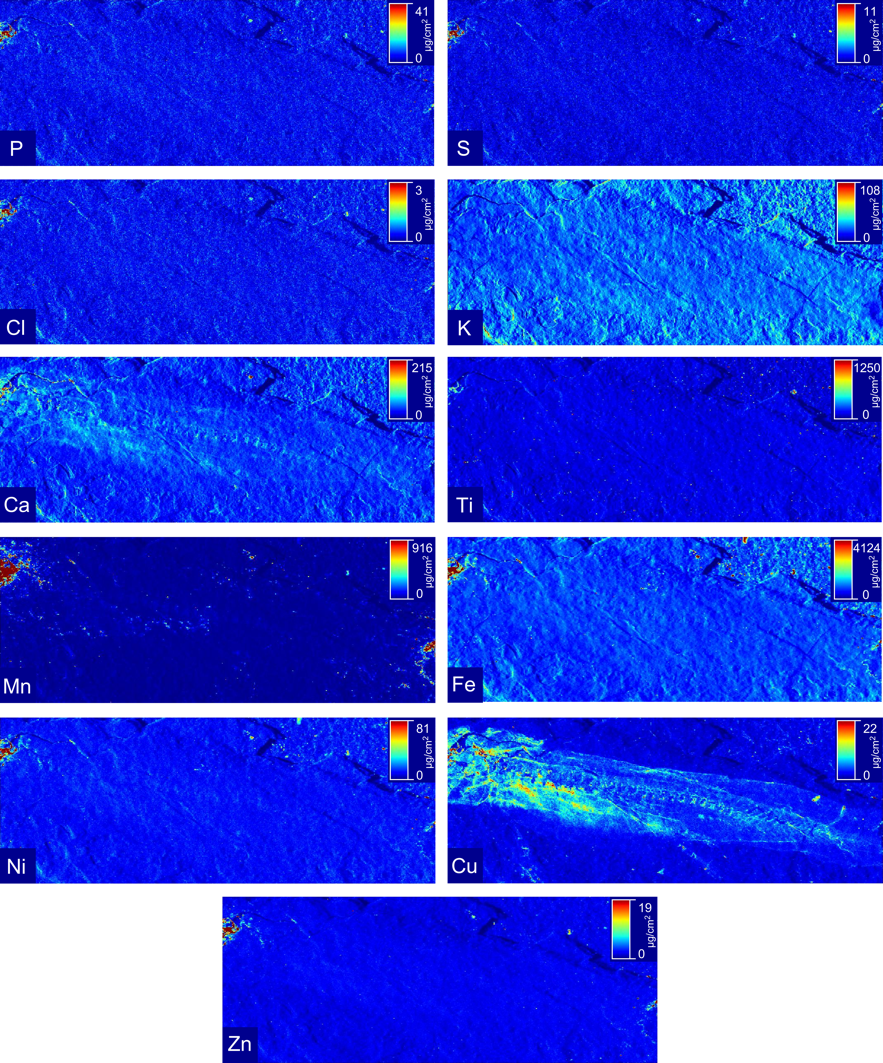


**Fig. S12** SRS**-**XRF maps of Amphibia indet*.* (CNU-SAL-NN2013002P) from Yanliao. Maps created using SMAK 1.50 <https://www.sams-xrays.com/smak>.

**
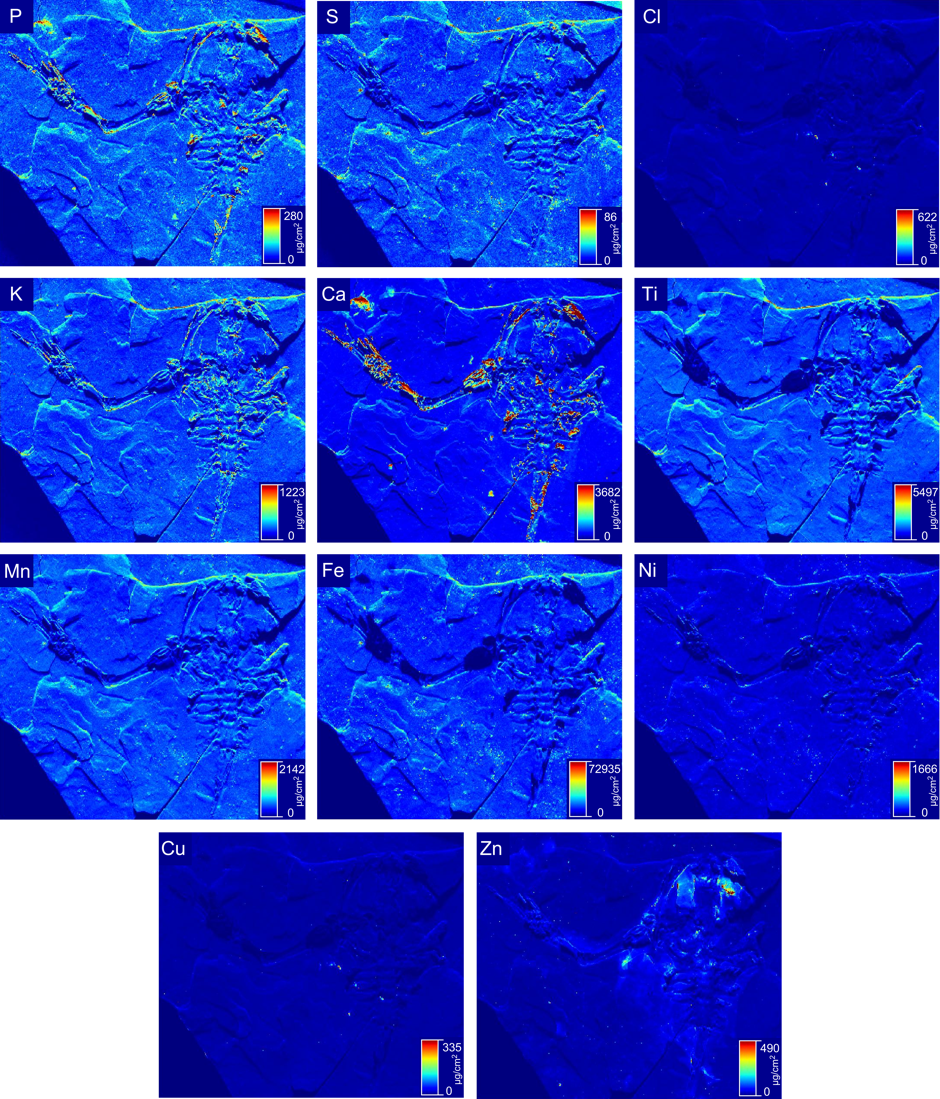
**

**Fig. S13** SRS**-**XRF maps of *Palaeobatrachus luedecki* (NHML-OR35814) from Bohemia. Maps created using SMAK 1.50 <https://www.sams-xrays.com/smak>.

**
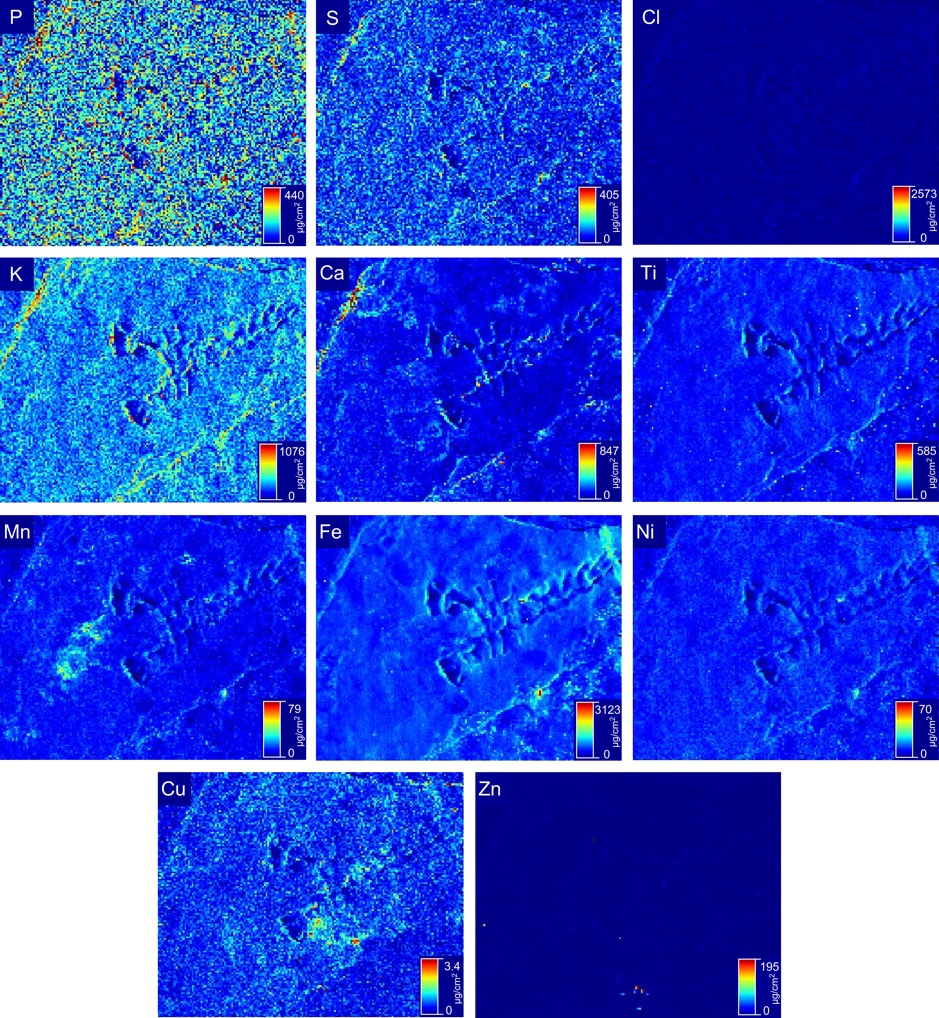
**

**Fig. S14** SRS**-**XRF maps of *Eopelobates sp.* (NHMB-Am908) from Orsberg. Maps created using SMAK 1.50 <https://www.sams-xrays.com/smak>.

**
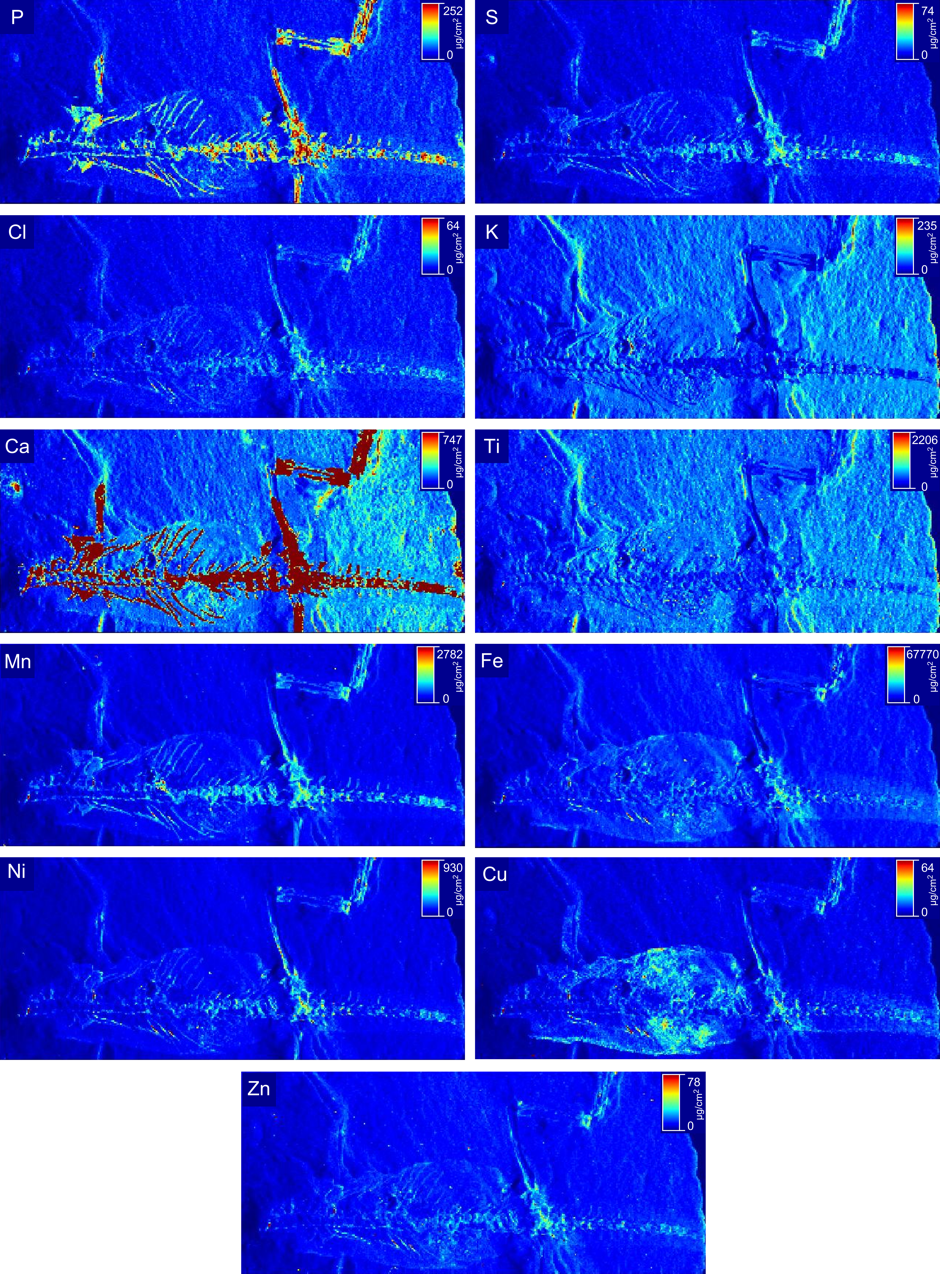
**

**Fig. S15** SRS**-**XRF maps of the reptile (Squamata indet., CNU-VER-LB2009001) from Yanliao. Maps created using SMAK 1.50 <https://www.sams-xrays.com/smak>.

**
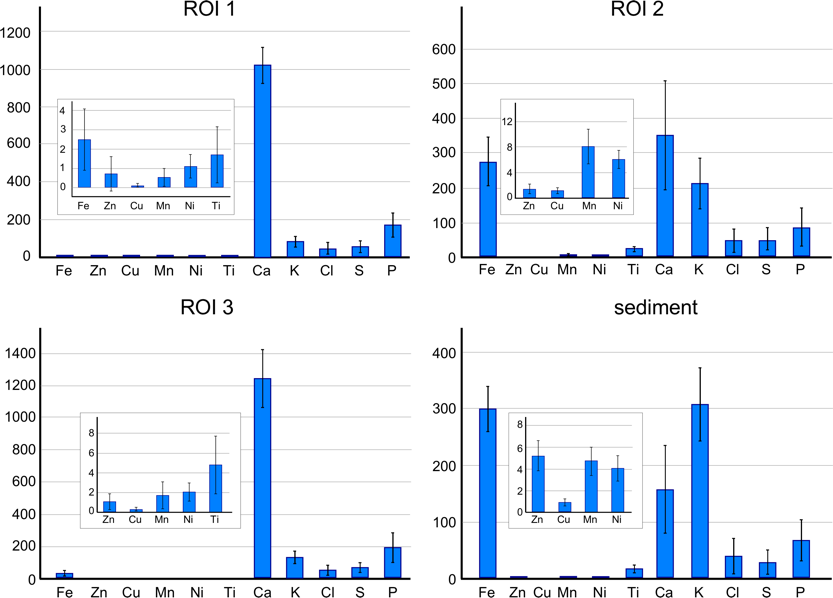
**

**Fig. S16** SRS**-**XRF data for *Neusticosaurus edwardsii* (PIMUZ-T3749). Units for Y-axis are μg/cm^2^.

**
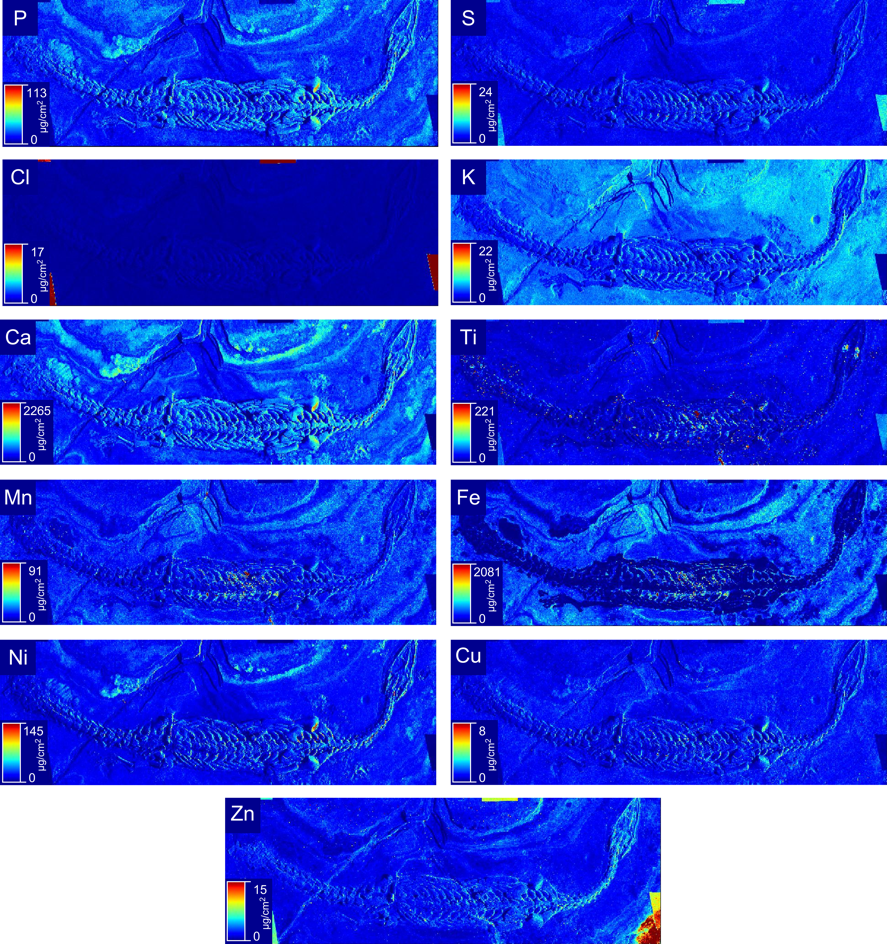
**

**Fig. S17** SRS**-**XRF maps of *Neusticosaurus peyeri* (PIMUZ-T3412) from Monte San Giorgio. Maps created using SMAK 1.50 <https://www.sams-xrays.com/smak>.

**
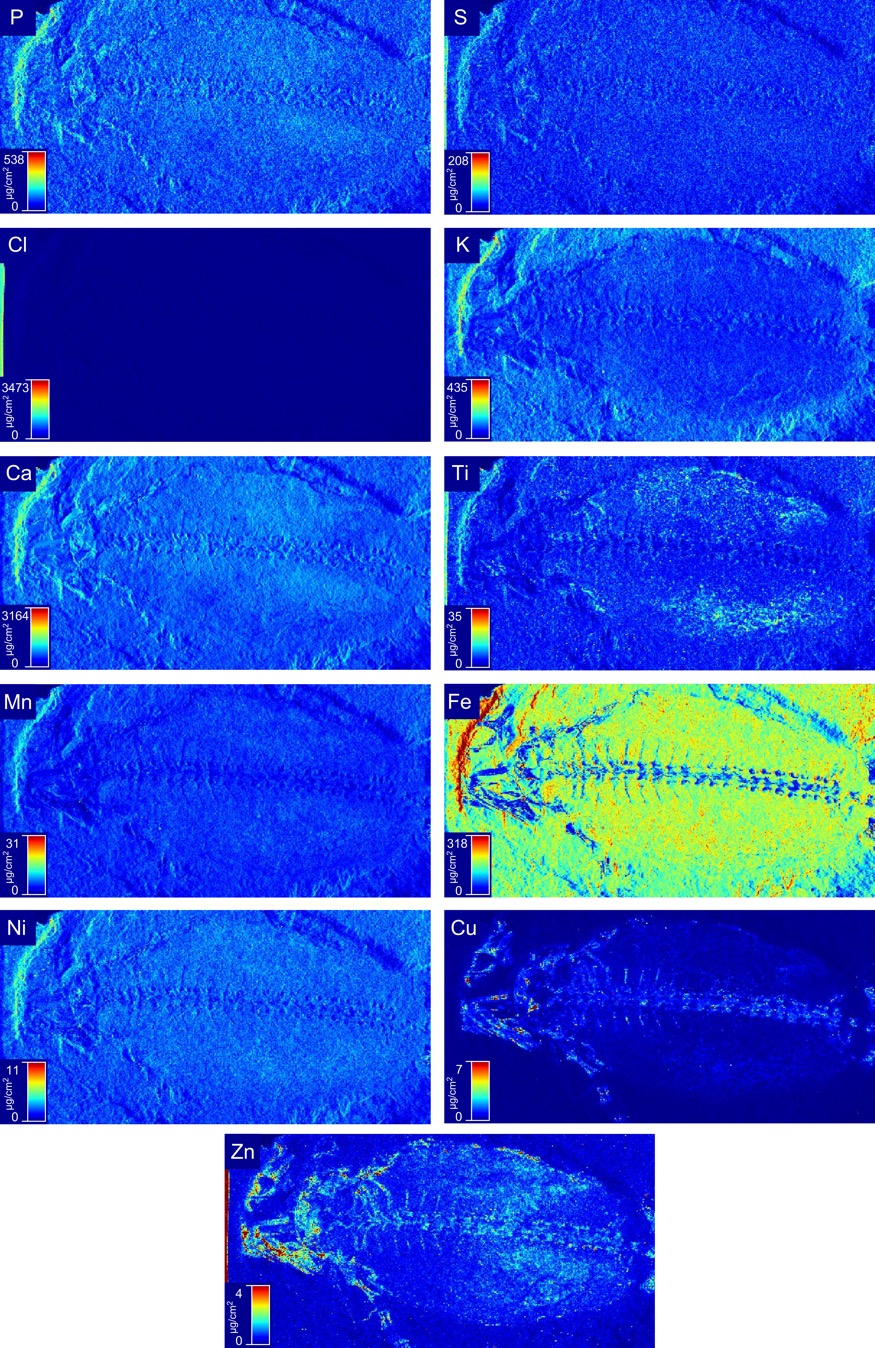
**

**Fig. S18** SRS**-**XRF maps of *Apateon pedestris* (NHMB-MB-Am 1220) from Saar-Nahe. Maps created using SMAK 1.50 <https://www.sams-xrays.com/smak>.

**
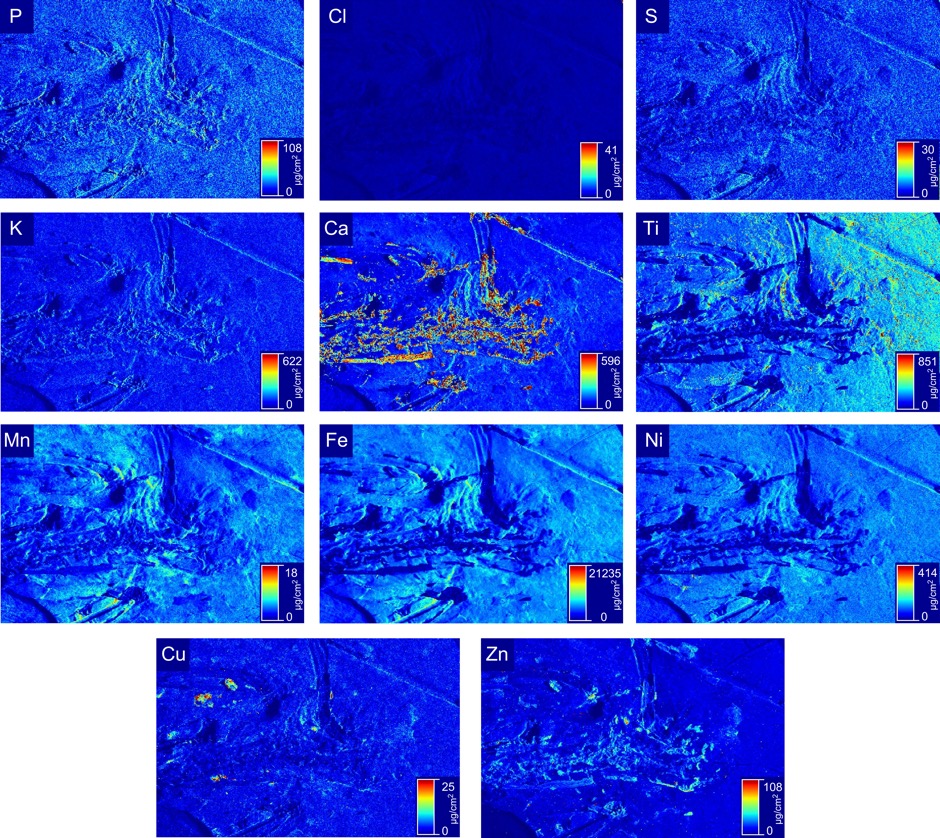
**

**Fig. S19** SRS**-**XRF maps of Aves indet. (HLMD-Me5472) from Messel. Maps created using SMAK 1.50 <https://www.sams-xrays.com/smak>.

**
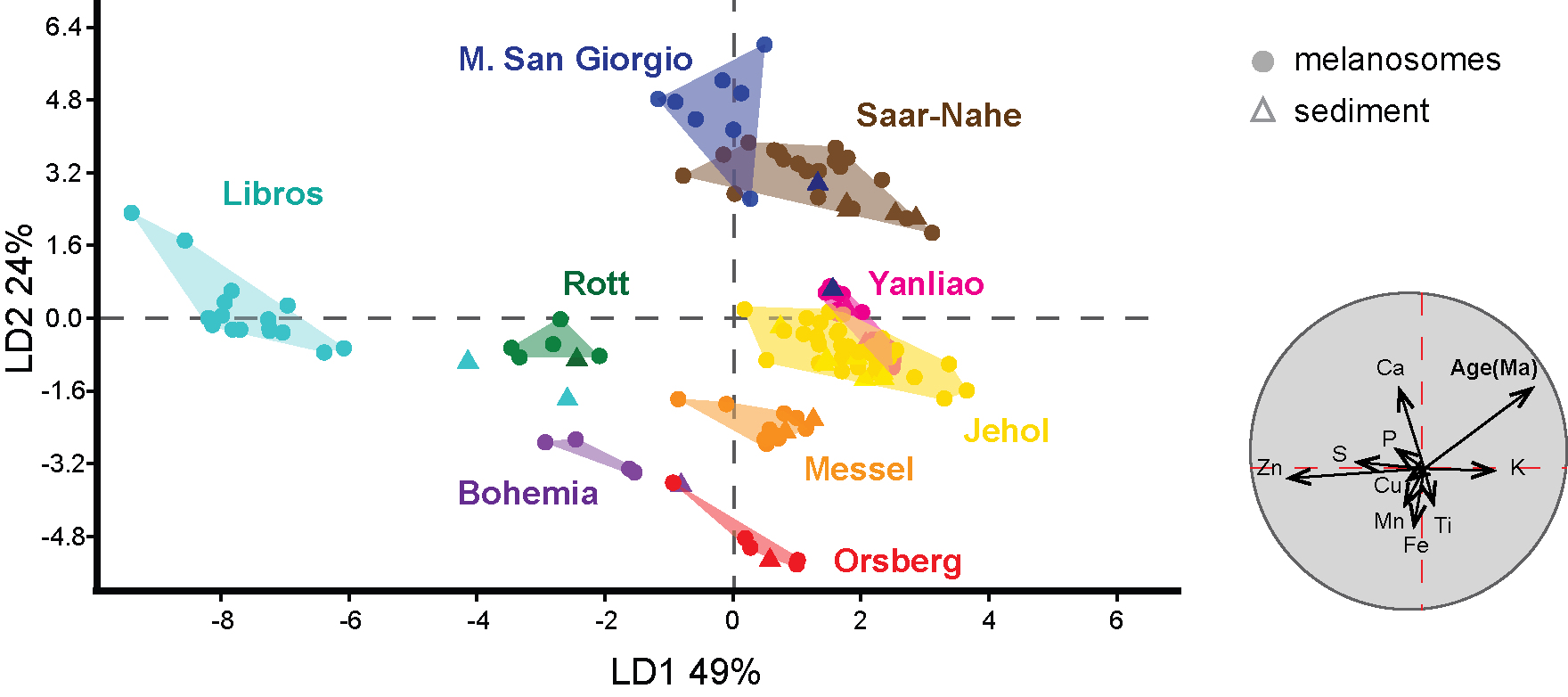
**

**Fig. S20** Linear Discriminant Analysis (LDA) of inorganic chemistry data and the age of the biotas for fossil melanosomes and sedimentary matrices. Scatterplot of the LDA chemospace for the entire dataset with biplot (grey circle). Biplots show the most discriminating variables, i.e. those that contribute most to the separation among groups.

**
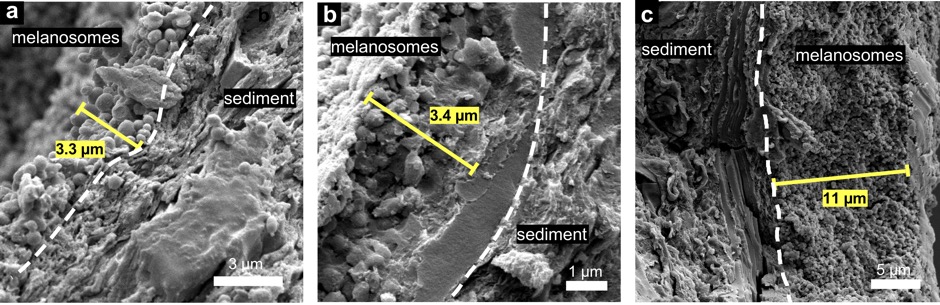
**

**Fig. S21** Scanning electron micrographs illustrating variations in the thickness of melanosome layers in different melanosome-bearing soft tissues. (**a**, **b**) Skin in *P. diluvianus* (NHML-30271) and in *P. luedecki* (NHML-OR35814). (**c**) Eyespot in *P. luedeki*.

**
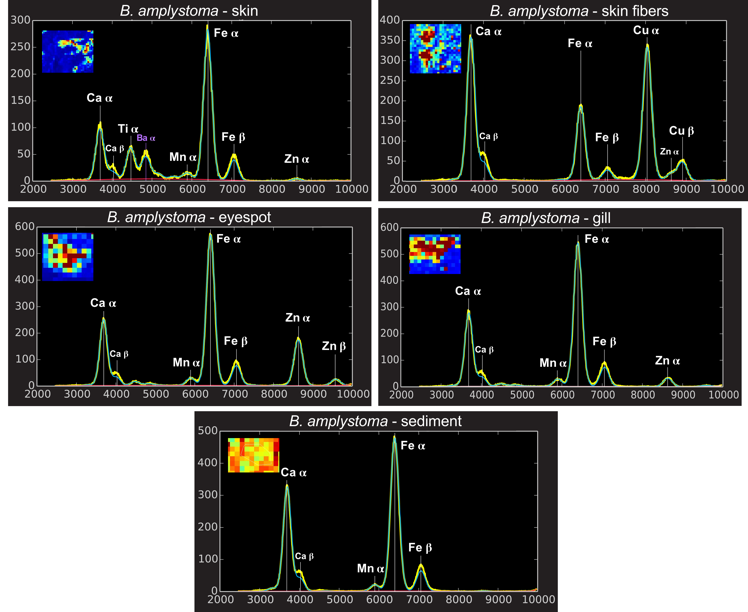
**

**Fig. S22** MCA spectra for ROIs from *B. amplystoma* (NHMD-155208). X-axis represents X-ray emission energy in eV; Y-axis represents counts. Yellow line represents the SRS-XRF spectrum of the sample of interest, blue line represents the fit, red line represents the continuum. MCA spectra created using SMAK 1.50 <https://www.sams-xrays.com/smak>.

**
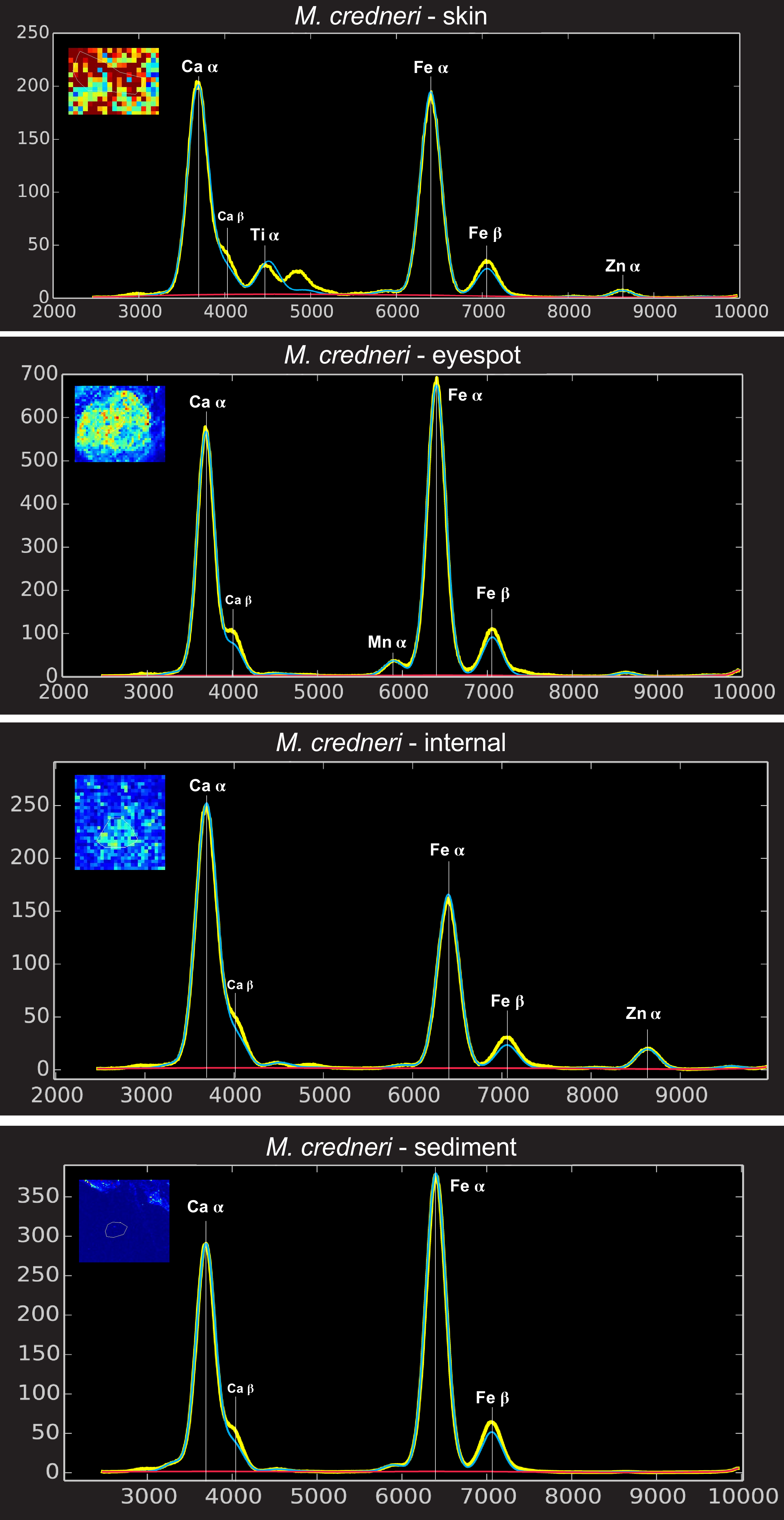
**

**Fig. S23** MCA spectra for ROIs from the amphibian *M. credneri* (NHMB-MB-Am.-1187). X-axis represents X-ray emission energy in eV; Y-axis represents counts. Yellow line represents the SRS-XRF spectrum of the sample of interest, blue line represents the fit, red line represents the continuum. MCA spectra created using SMAK 1.50 <https://www.sams-xrays.com/smak>. MCA spectra created using SMAK 1.50 <https://www.sams-xrays.com/smak>.

**
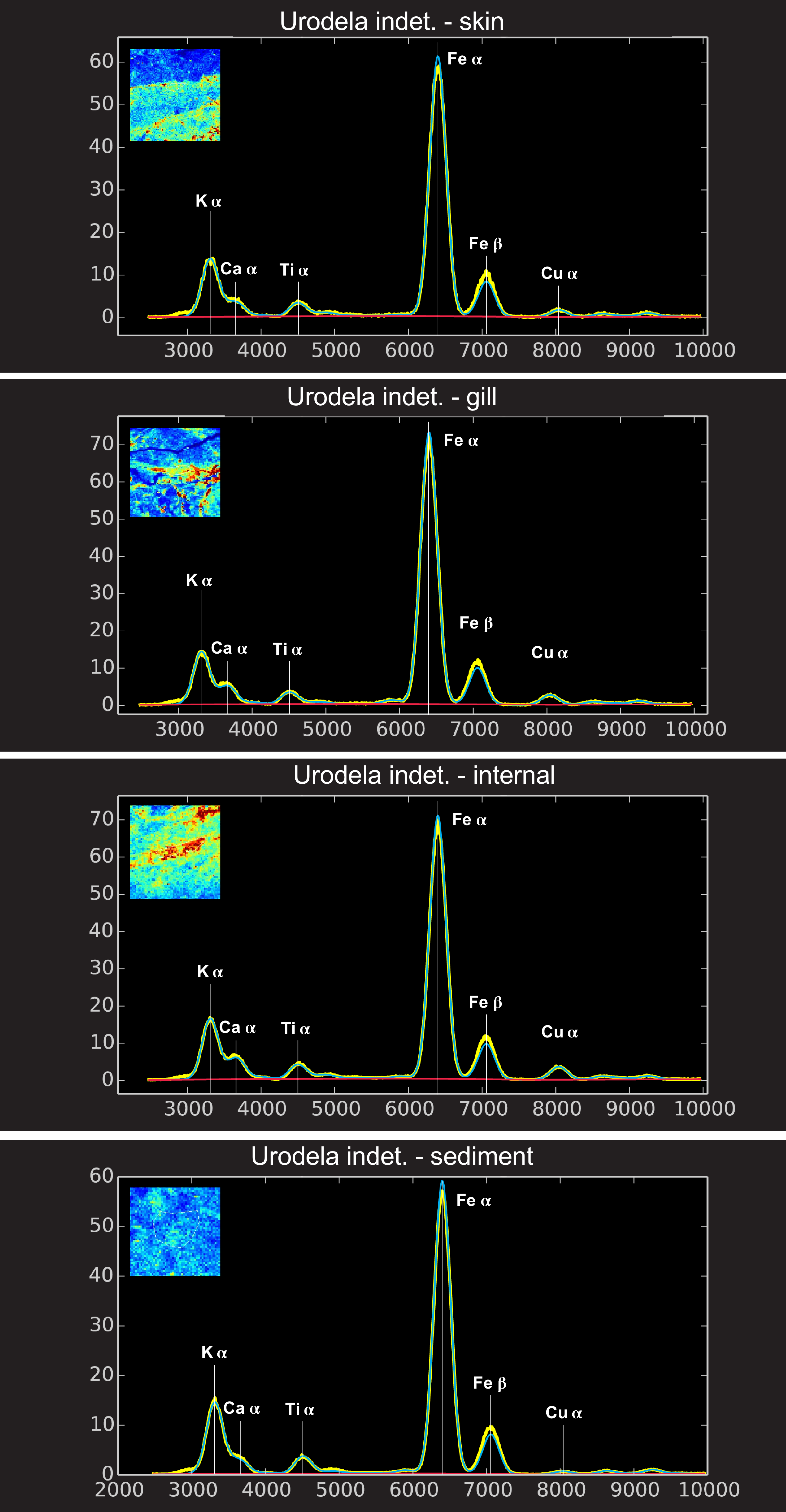
**

**Fig. S24** MCA spectra for ROIs from the fossil salamander (CNU-SAL-NN2013002P, Urodela indet.). X-axis represents X-ray emission energy in eV; Y-axis represents counts. Yellow line represents the SRS-XRF spectrum of the sample of interest, blue line represents the fit, red line represents the continuum. MCA spectra created using SMAK 1.50 <https://www.sams-xrays.com/smak>.

**
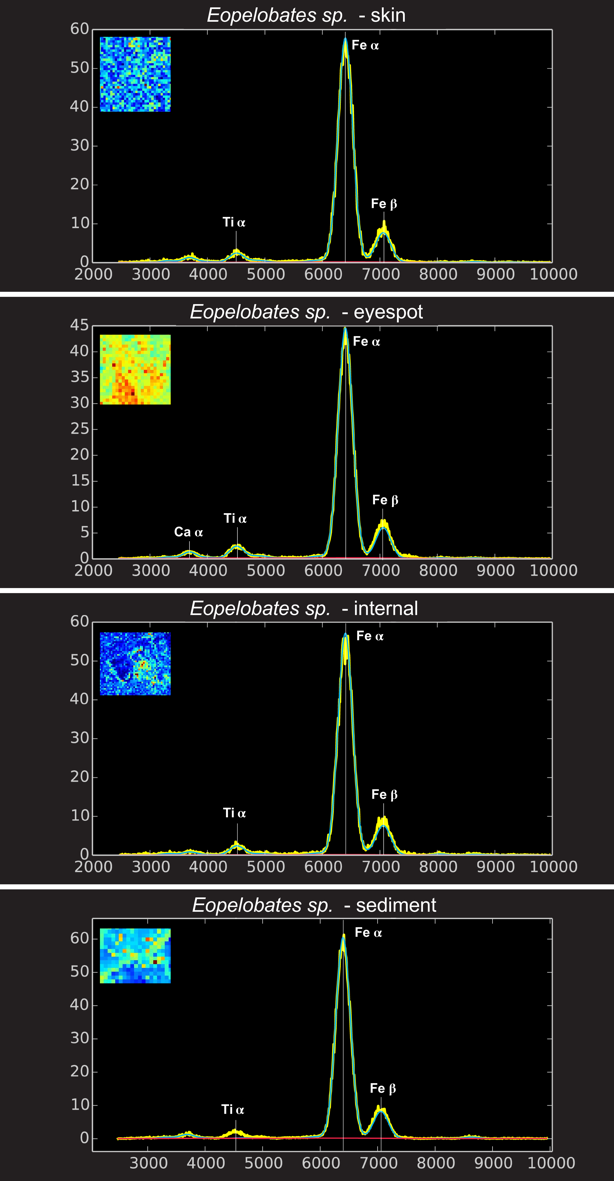
**

**Fig. S25** MCA spectra for ROIs from *Eopelobates sp*. (NHMB-Am908). X-axis represents X-ray emission energy in eV; Y-axis represents counts. Yellow line represents the SRS-XRF spectrum of the sample of interest, blue line represents the fit, red line represents the continuum. MCA spectra created using SMAK 1.50 <https://www.sams-xrays.com/smak>.

**
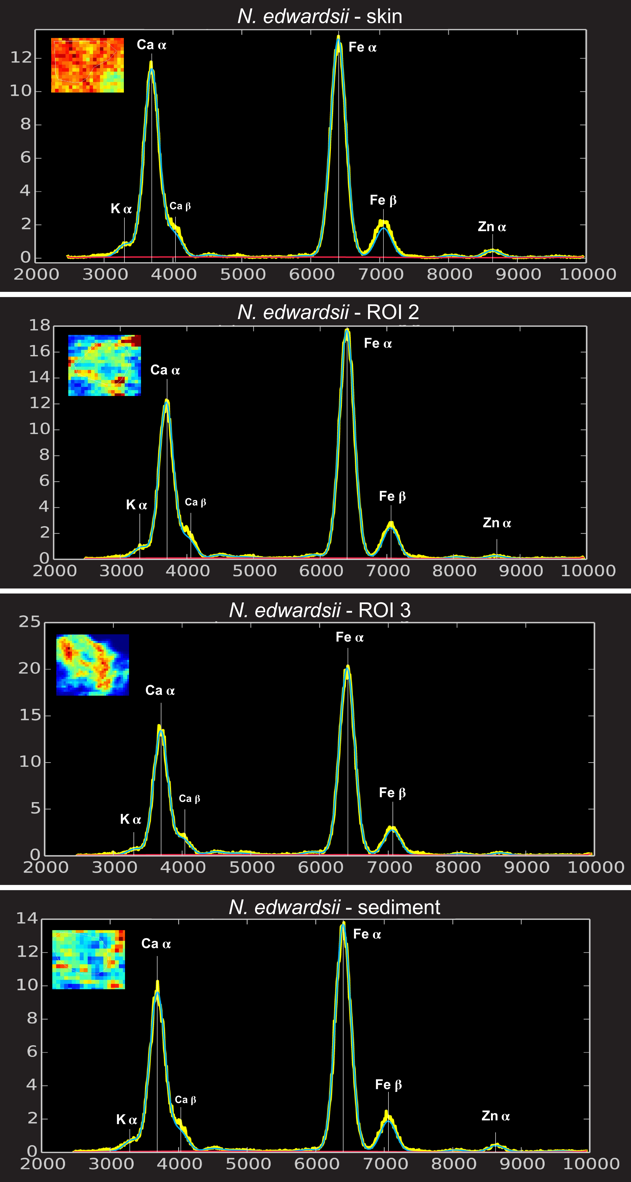
**

**Fig. S26** MCA spectra for ROIs from *N. edwardsii* (PIMUZ-T3749). X-axis represents X-ray emission energy in eV; Y-axis represents counts. Yellow line represents the SRS-XRF spectrum of the sample of interest, blue line represents the fit, red line represents the continuum. MCA spectra created using SMAK 1.50 <https://www.sams-xrays.com/smak>.

**
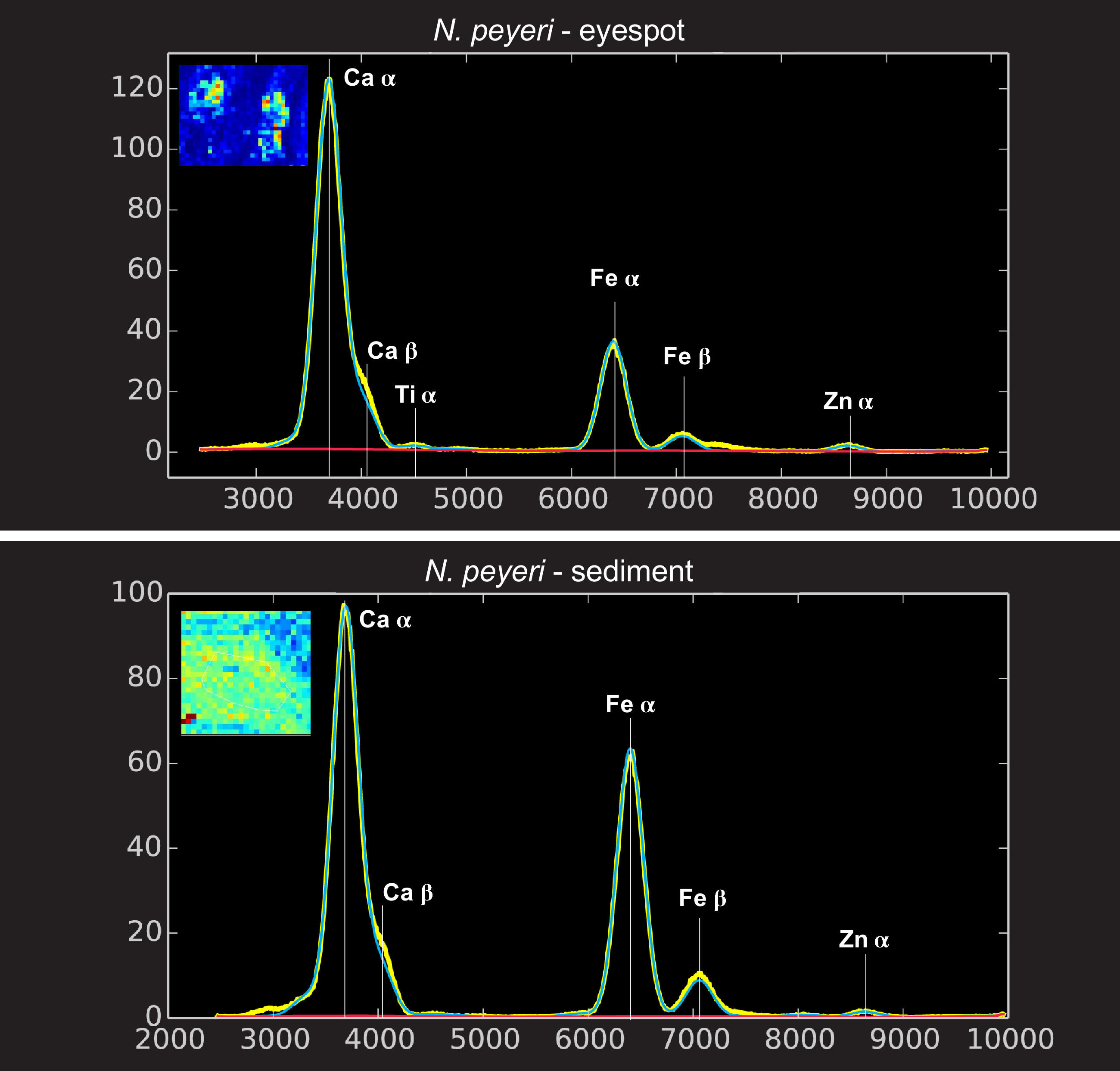
**

**Fig. S27** MCA spectra for ROIs from *N. peyeri* (PIMUZ-T3412). X-axis represents X-ray emission energy in eV; Y-axis represents counts. Yellow line represents the SRS-XRF spectrum of the sample of interest, blue line represents the fit, red line represents the continuum. MCA spectra created using SMAK 1.50 <https://www.sams-xrays.com/smak>.

**Supplementary Data legend**

**Supplementary Data S1.** SRS**-**XRF data (mean and standard deviation (StDev)) from soft tissue regions of interest and associated sedimentary matrix.

**Supplementary Data S2.** Raw SRS-XRF data from soft tissue regions of interest and sediments in fossil vertebrates.

**Supplementary Data S3. a.** LDA loadings for the inorganic chemistry of melanosomes and associated sediment (data organised by biota). **b.** LDA loadings for the analysis of the inorganic chemistry of melanosomes and sediments (data organised by taxon).

**Supplementary Data S4. a.** ANOVA and Kruskal-Wallis (*) tests. Bold text represents statistically significant p-values. **b.** Tukey HSD post-hoc and Mann-Whitney (*) tests. Bold text represents statistically significant p-values.

**Supplementary Data S5**. Mean values for SRS-XRF data for melanosome inorganic chemistry of fossil and extant amphibian soft tissues. These data are shown in the LDA and PCA plots in Fig. 7.

**Supplementary Data S6. a.** LDA loadings for the inorganic chemistry of melanosomes in extant and fossil amphibian species. **b.** PCA loadings for the inorganic chemistry of melanosomes in extant and fossil amphibian soft tissues.

**Supplementary Data S7.** Multi-channel analysis fit values for each analysed region.

**References**

1. McDiarmid, R. & Altig, R. *Tadpoles: the Biology of Anuran Larvae*. (University of Chicago Press, 1999).
